# Systemic immune activation coexists with compartment-specific regulatory enrichment in matched blood, peritumor, and tumor tissue from advanced oral squamous cell carcinoma

**DOI:** 10.3389/fimmu.2026.1923358

**Published:** 2026-10-01

**Authors:** Nayane Alves de Lima Galdino, Flávio Pignataro-Oshiro, João Figueira Scarini, Raísa Sales de Sá, Guilherme Ferreira de Britto Evangelista, Amanda Braga Figueiredo, Clara Maciel Cavalcanti, Katia Luciano Pereira Morais, Vanessa Alves Gasparini, Hugo Fontan Kohler, Thiago Bueno de Oliveira, Luiz Paulo Kowalski, Kenneth J. Gollob

**Affiliations:** 1 Translational Immuno-Oncology Group, International Research Center, A.C.Camargo Cancer Center, São Paulo, SP, Brazil; 2 Translational Immuno-Oncology Laboratory, Faculdade Israelita de Ciências da Saúde Albert Einstein, Hospital Israelita Albert Einstein, São Paulo, SP, Brazil; 3 Center for Research in Immuno-Oncology (CRIO), Faculdade Israelita de Ciências da Saúde Albert Einstein, Hospital Israelita Albert Einstein, São Paulo, SP, Brazil; 4 Flow Cytometry Core Facility, Memorial Sloan-Kettering Cancer Center, New York, NY, United States; 5 International Research Center, A.C.Camargo Cancer Center, São Paulo, SP, Brazil; 6 Medical Oncology Department, Head and Neck Cancer Reference Center, A.C.Camargo Cancer Center, São Paulo, SP, Brazil; 7 Department of Head and Neck Surgery and Otorhinolaryngology, A.C.Camargo Cancer Center, São Paulo, SP, Brazil; 8 Head and Neck Surgery Department, Medical School, University of São Paulo, São Paulo, SP, Brazil

**Keywords:** advanced OSCC, high-dimensional immune profiling, immune compartments, immune exhaustion, MAIT-like cells, PD-1/PD-L1, tumor microenvironment

## Abstract

**Background:**

Oral squamous cell carcinoma (OSCC) is the most prevalent form of oral cancer and is characterized by a poor prognosis and frequent immune evasion. While the tumor microenvironment (TME) plays a central role in modulating responses to therapy, its relationship with systemic immune signatures remains poorly understood.

**Methods:**

We performed high-dimensional immune profiling in matched peripheral blood, adjacent non-neoplastic peritumor mucosa, and tumor tissue from Human Papillomavirus (HPV)-negative, treatment-naïve patients with advanced OSCC. Soluble mediators were quantified by multiplex immunoassay, and immune cell phenotypes were assessed by multiparametric flow cytometry. Compartment-specific patterns were evaluated using paired and unpaired comparisons, dimensionality reduction, ordinal trend analysis, unsupervised clustering, and correlation networks.

**Results:**

Seventeen plasma mediators were significantly elevated in OSCC patients compared with healthy donors. Paired cellular profiling identified compartment-specific differences across blood, peritumor and tumor, and selected PD-1^+^CD95^+^, CD25^+^FoxP3^+^, and PD-L1^+^ populations showed significant progressive trends along the blood-peritumor-tumor axis. CD4^+^ mucosal-associated invariant T (MAIT)-like cells (TCR Vα7.2^+^CD161^+^) were relatively enriched in peritumor tissue, but the peritumor compartment was not uniformly intermediate across all populations. Correlation networks showed limited overlaps, with greater overlap between peritumor and tumor networks than between either tissue compartment and blood. Plasma profiling showed elevated pro-inflammatory cytokines, chemokines, and growth factors, consistent with systemic immune activation. Peripheral blood exhibited an activated phenotype with increased frequencies of activated T and NK cells, alongside CD4^-^CD8^-^ TCR Vα7.2^+^CD161^+^ cells. In contrast, the tumor microenvironment was enriched for CD95^+^PD-1^+^ CD4^+^ and CD8^+^ T cells, NK cells and PD-L1^+^ monocytes and CD4^+^ MAIT-like cells. The peritumor compartment showed a distinct immune profile marked by regulatory T cell accumulation and other immunosuppressive mediators, rather than a uniform intermediate state between blood and tumor. Correlation networks revealed pronounced shifts in immune coordination across compartments.

**Conclusions:**

Advanced OSCC is associated with distinct systemic and tissue immune profiles. These exploratory findings identify compartment-specific immune features and selected monotonic trends that warrant future spatial, functional, and clinical validation.

## Introduction

Oral squamous cell carcinoma (OSCC) represents a growing global public health issue. In 2024, cancers of the lip and oral cavity accounted for an estimated 452,205 new cases and 194,108 deaths worldwide, with these numbers projected to reach approximately 664,845 cases and 309,110 deaths by 2050 (33). Risk factors such as tobacco, alcohol and betel quid consumption, and human papillomavirus (HPV) infection contribute to the development of oral potentially malignant disorders, which are prone to progress to OSCC (1). OSCC arises predominantly from squamous cells lining the mouth and accounts for over 90% of oral cancers. Other oral cancers, including salivary gland malignancies, sarcomas and melanomas, constitute less than 10%, with about 1% of metastatic cases originating from the lungs, breasts, prostate, and kidneys. OSCC can vary in differentiation and often metastasizes to lymph nodes, with spread related to T classification, depth of invasion, and lymphovascular invasion (2).

OSCC can be detected early, but many cases are diagnosed at an advanced stage, necessitating systemic therapy to improve local control and disease-specific survival (3–5). Nevertheless, many patients experience poor outcomes, largely due to late diagnosis and the limited effectiveness of current therapies (4). Despite advancements in diagnostic and treatment methods, the global survival rate for oral cancer remains poor, highlighting the urgent need for novel therapeutic approaches (6). One of the primary challenges in treating oral cancer is its complex molecular biology and intricate interaction with the tumor microenvironment (TME). The TME encompasses a diverse array of stromal cells, immune cell subpopulations, and signaling molecules, which play a crucial role in cancer progression and resistance to treatment. The TME can induce an immunosuppressive response that hampers antitumor immunity and contributes to tumor aggressiveness, underscoring the importance of immune checkpoints and the microenvironment’s role in regulating immune responses (7–10).

The role of the tumor microenvironment in cancer development and its impact on clinical outcomes has increasingly been recognized as a critical component of overall disease dynamics. In some cases, it has been shown to be a determinant in tumor progression, therapeutic response, and recurrence (11). Elucidating the immunological mechanisms related to clinical outcomes may facilitate the development of novel immunotherapies and the identification of biomarkers capable of predicting prognosis or response to specific treatments (12). This would enable targeted and effective use of therapies, thereby reducing costs and improving the likelihood of treatment success and survival. Despite advancements in research regarding the complexities of TME interactions in the context of OSCC, these dynamics are not yet fully understood. Notably, our analysis offers an integrated approach by assessing both soluble immune profiles at diagnosis and three cellular immune compartments (blood, peritumor and tumor) from the same patients.

A major breakthrough in the treatment of advanced-stage head and neck cancer was the addition of perioperative pembrolizumab. A clinical trial demonstrated improved event-free survival when compared to standard treatment with pathological response in a significant proportion of patients (13), suggesting that treatment aimed at immune checkpoints may improve outcomes for these patients.

Here, we focused on the characterization of local and systemic immune profiles of treatment-naive patients with advanced-stage OSCC by integrating soluble mediators and cellular phenotypes across blood, peritumor and tumor. By comparing matched compartments from the same patients, this exploratory analysis seeks to define compartment-specific immune patterns and generate hypotheses for future mechanistic and clinical validation.

## Materials and methods

### Patient recruitment

All patients received care at the Department of Head and Neck Surgery and Otorhinolaryngology at A.C.Camargo Cancer Center, São Paulo, Brazil. This study was a prospective, non-interventional investigation. Patients were assigned to specific therapeutic strategies (surgery with or without postoperative radiotherapy or chemotherapy) in accordance with the hospital’s standard clinical practice. Diagnosis, treatment, and patient follow-up were conducted independently of the decision to include patients in this study. The study focused on patients with stage III and IV OSCC to specifically target those with locally advanced disease. In addition, all patients were treatment-naïve, HPV negative as determined by p16 immunohistochemistry, and all primary tumors arose in the oral cavity. p16 positivity was defined as moderate-to-strong nuclear and cytoplasmic staining in 70% or more of tumor cells (14, 15). Healthy individuals (n = 7) were included as a control group, following the selected inclusion criteria: Adult individuals without cancer or oral potentially malignant disorders, chronic or immunosuppressive diseases, or under immunosuppressive treatments. All were nonsmokers and informed only of social alcohol consumption, if any. Sample processing, staining and analysis followed the same procedures as the study group, except for tissue collection, which did not happen. All recruited patients provided written informed consent in accordance with the requirements of the Brazilian National Research Ethics Committee, and the study was approved by A.C.Camargo's Research Ethics Commitee Research Ethics Committee (protocol no. 2532/18B).

### Inclusion and exclusion criteria

Adult individuals (aged 18 to 90 years) diagnosed with OSCC and treated at A.C.Camargo Cancer Center were included. Patients with OSCC of the tongue (excluding the base), gums, floor of the mouth, buccal regions, and hard palate were included. Patients were classified according to the International Classification of Diseases for Oncology, 3rd Edition, 1st Revision (ICD-O-3.1), with topographies C02-C06 (16). Patients with previously treated or synchronous cancers, and those using immunosuppressive agents were excluded. Patients with distant metastasis, debilitating chronic diseases, autoimmune diseases, or chronic infections were also excluded.

### Sample collection

Peripheral blood was collected into two 4 mL EDTA tubes before initiation of any oncologic treatment. Blood was collected, in median, eight days before the surgery (range: 0 to 43 days). Blood samples were centrifuged (400xg/10 min/25 °C) and plasma separated and stored at -80 °C. The remaining blood volume was diluted with an equal volume of PBS, and peripheral blood mononuclear cells (PBMCs) were purified using a density gradient centrifugation protocol. A portion of the PBMCs (approximately 1x10^6^ cells/mL) was analyzed. Fresh surgical specimens (tumor and peritumor tissues) were processed using the MediMachine (BD Biosciences). A portion (1x10^6^ cells/mL) was cryopreserved, and another portion was used for immune profiling of fresh tissue via flow cytometry. Peritumor tissue comprised an approximately 1 cm³ specimen that was pathologically assessed as tumor-free. The specific site of removal was left to the discretion of the attending pathologist but comprised the closest uncompromised tissue relative to the tumor, as confirmed by frozen section biopsy. This could represent tissue medial, lateral, anterior or posterior to the primary tumor. The peritumor tissue was further evaluated to exclude the presence of oral potentially malignant disorders (OPMDs) or microscopic tumor contamination, while degrees of inflammation and dysplasia were recorded individually. Sampling of areas affected by necrosis was avoided in both tumor and peritumor specimens.

### Immune profiling by multiparametric flow cytometry

Immune cell profiles were characterized using multiparametric flow cytometry (FACSymphony A5, BD Biosciences). T lymphocytes (T_H_1 [CD4^+^T-bet^+^], cytotoxic T [CD8^+^Granzyme B^+^], and Treg [FoxP3^+^]), B lymphocytes (CD19^+^), NK (CD56^+^) and NKT (CD56^+^CD4^+^ or CD56^+^CD8^+^) cells, along with their activation/inhibition markers (e.g., CD161, CD25, PD-1, CD95), cytolytic molecules (Granzyme B), and immunological memory states (naïve, revertant, central memory, and effector memory, based on CCR7 and CD45RO expression) were assessed. Myeloid cell populations, including monocytes (classical, intermediate, and non-classical), macrophages, and dendritic cells, were evaluated with specific surface markers (e.g., HLA-DR, CD38, CD14, CD16, CD11b, CD11c, PD-L1, and PD-L2). FVS575 was added to the panel to assess cell viability. Panels were validated prior to the study to generate compensation matrices and instrument settings for each sample. Analyses were conducted using cells from surgical specimens (tumor and peritumor tissue) and peripheral blood collected at baseline. Cell viability was confirmed using trypan blue exclusion, and cells were resuspended in staining solution with the appropriate antibody panels (**Supplementary Table 1**). Intracellular markers were assessed by fixing, permeabilizing, incubating with intracellular antibodies, washing, and acquiring data on the FACSymphony A5. At least 50,000 events were acquired for each analysis when possible. Mean counts per cell in each panel are detailed in the **Supplementary Material** (**Supplementary Table 2**). Data were analyzed using FlowJo v. 10.9 software (BD Biosciences) and presented as frequency (%) of parent population. A complete gating strategy and representative plots are presented in the **Supplementary Material** (**Supplementary Figures 1**, **2**). Unsupervised analysis was performed with at least 2,000 events per sample. For Panel 1, cells were sequentially gated on live cells, CD45^+^ cells, and the lymphocyte population based on FSC × SSC characteristics. Next, data resolution reduction was performed with *Downsample* v3.3 plugin. The resulting events were analyzed using the phenotypic parameters included in the panel, including CD3, CD4, CD8, CD56, PD-1, CD95, CD161, GZMB, FoxP3, and other relevant markers. For Panel 2, cells were sequentially gated on live cells and CD45^+^ cells, followed by identification of the lymphocyte and monocyte populations based on FSC × SSC characteristics. *Downsample* plugin was subsequently employed within these gated populations. The selected events were analyzed according to the phenotypic parameters included in the panel, including CD11b, CD11c, CD19, PD-L1, and other relevant markers. Data from distinct samples were concatenated for further analysis for each panel individually. A Uniform Manifold Approximation and Projection (UMAP, plugin version 4.1) was used to reduce dimensionality and visualize the distributions of events using the phenotypic parameters selected for each panel, while the PhenoGraph v4.05 algorithm was used to identify the major cell populations based on marker expression (17, 18). UMAP (v4.1) settings were Euclidean distance, 15 nearest neighbors, 0.5 minimum distance, and number of components set to 2. PhenoGraph was employed with K = 30 and Run ID = auto. Samples were manually annotated according to marker’s mean fluorescence intensity histogram plots.

### Flow cytometry controls

To reduce batch effects from samples gathered across different months we employed both Cytometer Setup and Track (CST) beads and Rainbow Beads to guarantee that each sample was read with similar conditions. Each antibody was titrated from the manufacturer’s recommended concentration to the best usable concentration optimized per cell subset of interest. This assessment used the stain index as a basis, aside from comparative visual plots to better segregate negative and positive populations. A pool of cells from fresh samples available by collection day was used for compensation and served as a negative-stained control. For panel one, which had intracellular and extracellular markers, the negative-stained control went through all the fixation and permeabilization procedures. Lastly, we further opted for staining fresh samples instead of conducting experiments in large batches to reduce the effects of freezing and thawing processes in cell viability and marker expression.

### Soluble immune profile assessment by multiplex immunoassay

Plasma from patients and healthy subjects was evaluated for 48 inflammatory and anti-inflammatory analytes using the Bio-Plex Pro Human Cytokine 48-Plex Immunoassay kit on the Bio-Plex^®^ 200 system (BioRad Inc., USA). Analytes included cytokines, chemokines, and growth factors. Plasma samples were incubated with a bead mixture specific to each analyte, followed by detection with a fluorescently labeled antibody. Analyte concentrations (pg/mL) were calculated using a standard curve.

### Statistical analysis

Statistical analyses were conducted using GraphPad Prism software, version 8.3. Data normality was assessed using the Kolmogorov-Smirnov test. Comparisons among blood, peritumor, and tumor samples were made using ANOVA for normally distributed data or the Kruskal-Wallis test for non-normally distributed data, with respective *post hoc* tests (Holm-Sidak or Dunn). For the subset of patients with all three matched compartments, paired comparisons across blood, peritumor, and tumor were performed using the Friedman test with Dunn’s *post hoc* correction. An ordinal trend analysis was done with Page’s L trend tests using a code designed by Grossmann on RStudio (Posit Software) version 2024.12.0 (**Supplementary File 1**). Furthermore, Line Trend analysis was done with a built-in tool from Microsoft Excel (Microsoft 365). The unpaired Kruskal-Wallis analysis was retained as a secondary comparison to incorporate the full set of available samples and is presented in the **Supplementary Material**. Comparisons between two groups were made using Student’s t-test or the Mann-Whitney U-test, depending on data distribution. All p-values were assessed with Benjamini-Hochberg False Discovery Rate (FDR) test" at a 5% error rate, and adjusted p-values below 0.05 were considered statistically significant. Heatmaps were generated using the online ClustVis tool (19), after data transformation by Ln(x+1). Euclidean was used as a distance metric, and Ward was the chosen method of linkage. Principal Component Analysis (PCA) biplot and related graphs were generated using the *factoextra* and *FactoMineR* packages on RStudio (Posit Software) version 2024.12.0. Multiple correlation analysis and matrices were performed using the *corrplot* package on RStudio (Posit Software) version 2024.12.0, using Spearman’s correlation and considering only factors that had an FDR-adjusted p-value below 0.05 in previous comparisons. All Spearman’s correlations which resulted in a p-value below 0.05 were used to construct correlation matrices and networks, where distinct p-value levels are highlighted with different notations. For matrices, correlations with p-values above 0.05 are not highlighted, while in correlation networks those correlations were ignored. Correlation networks were constructed using Cytoscape version 3.10.3. Factors were color-coded according to similar functions or characteristics, and sphere size was set to reflect the number of interactions made by each factor (**Supplementary Table 3**). Edges were colored blue for inverse correlations and red for direct correlations. Overlap analysis was performed by searching for interactions shared by distinct groups, where interactions with opposite coefficient values (e.g., positive and negative) were considered not shared. Overlap percentage represents the number of shared interactions divided by the total number of interactions in both networks. Interactions that are not shared were deemed “exclusive”. The percentage of interactions was calculated by grouping factors and assessing the mean number of interactions. In some cases, the matrix and network were constructed using the same populations (e.g., CD4 MAIT-like lineage cells) present in blood, tumor and peritumor. Raw p-values and, where applicable, Benjamini-Hochberg False Discovery Rate FDR-adjusted p-values found across all analyses are reported in the **Supplementary online Material**. This study is exploratory, and no formal *a priori* power calculation was performed for the specific comparisons reported here. Our group typically designs flow cytometry and multiplex immunoassay studies for 80% power with ~20 subjects per group; however, the principal comparisons in this study are paired, within-patient contrasts across compartments (blood, peritumor, and tumor) in the same 17 patients, rather than between independent clinical groups, a design that requires substantially fewer subjects than an unpaired comparison to reach equivalent power, since within-patient correlation reduces the error variance. The unpaired comparison against healthy donors (n = 7) is smaller than our typical benchmark and is addressed as a limitation.

## Results

### Cohort profile

The study cohort comprised 25 patients (17 male, 8 female) with a median age at diagnosis of 60 years (range: 41–87). The most common tumor site was the tongue (44.00%), followed by the buccal mucosa (24.00%), hard palate (16.00%), and alveolar ridge (16.00%). Most patients were diagnosed at an advanced clinical stage, with 72.00% classified as pathological stage IV and 28.00% as pathological stage III. Regarding nodal involvement, 40.00% of patients presented with pN0 disease, while 24.00% had pN1, 20.00% had pN2, and 16.00% had pN3. In terms of tumor size, 16.00% of patients had pT2 tumors, 44.00% had pT3, and 40.00% had pT4. With respect to lifestyle factors, 16.00% of the patients were active smokers, and 20.00% reported regular alcohol consumption. These data are summarized in **Table 1**.

**Table 1:** Clinical and epidemiological characteristics of the study cohort.

Characteristics	n* (%)	
Oral squamous cell carcinoma patients		Healthy control
Age at diagnosis, median (range)	60 (41 – 87)	30 (22 – 60)
Sex		
Female	8 (32.00%)	2 (28.57%)
Male	17 (68.00%)	5 (71.43%)
Tumor site, n (%)		
Tongue	11 (44.00%)	Not Applicable
Buccal mucosa	6 (24.00%)	Not Applicable
Hard palate	4 (16.00%)	Not Applicable
Alveolar ridge	4 (16.00%)	Not Applicable
Pathological stage, n (%)		
III	7 (28.00%)	Not Applicable
IV	18 (72.00%)	Not Applicable
pN stage, n (%)		
N0	10 (40.00%)	Not Applicable
N1	6 (24.00%)	Not Applicable
N2	5 (20.00%)	Not Applicable
N3	4 (16.00%)	Not Applicable
pT stage, n (%)		
T2	4 (16.00%)	Not Applicable
T3	11 (44.00%)	Not Applicable
T4	10 (40.00%)	Not Applicable
Smoking status, n (%)		
Current smoker	4 (16.00%)	0 (0.00%)
Ex-smoker	5 (20.00%)	0 (0.00%)
Never smoke	15 (60.00%)	7 (100.00%)
NA	1 (4.00%)	0 (0.00%)
Drinking status, n (%)		
Regular drinker	5 (20.00%)	0 (0.00%)
Social drinker	6 (24.00%)	4 (57.14%)
Never drank	13 (52.00%)	3 (42.86%)
NA	1 (4.00%)	0 (0.00%)

*n = 25 patients and 7 healthy controls; NA, Not Available.

### OSCC patients exhibit an exacerbated systemic soluble immune profile compared to healthy individuals

To characterize systemic immune dysregulation in newly diagnosed, treatment-naïve OSCC patients, we evaluated the plasma concentrations of 48 circulating immune mediators. Plasma samples from 17 OSCC patients were analyzed and compared with samples from 7 healthy donors (HD), recruited as controls (**Figure 1**). In parallel, multiparametric flow cytometry was performed on peripheral blood mononuclear cell (PBMC) samples, along with paired tumor and peritumor tissue samples from 17 patients, enabling immune profiling across multiple anatomical compartments (**Figure 1A**). The number of samples per assay in our main (paired) analysis is detailed (**Figure 1B**). A secondary, unpaired analysis with all available samples was also performed and presented in the **Supplementary Material**.

**Figure 1:**
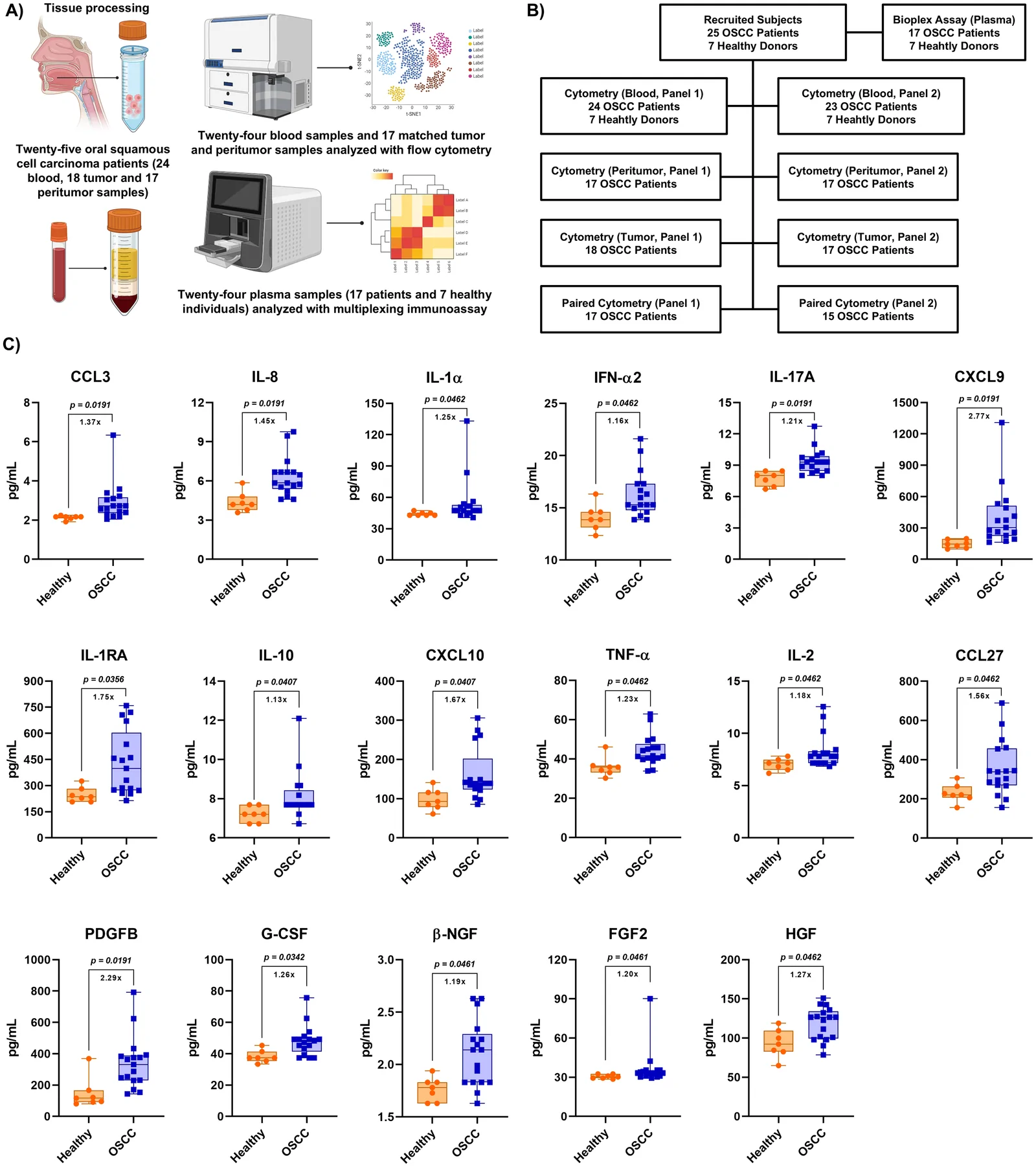
OSCC plasma shows broad upregulation of inflammatory cytokines and growth factors. Patients with stage III or IV oral squamous cell carcinoma (OSCC) were recruited and had blood drawn before the administration of any kind of treatment. Blood from healthy donors was used as a control. The plasma was separated and stored at -80 °C until the concentration of 48 factors was measured with a multiplex bead assay kit. **(A)** Schematic workflow showing sample collection, processing and analysis. **(B)** Flow chart representing sample distribution for each assessed assay and flow cytometry panel. **(C)** Graphs show the median concentration of multiple immune mediators in picograms/mL and their interquartile range and minimum and maximum values. The distributions of analyte concentrations between healthy individuals (n = 7) and OSCC patients (n = 17) were compared using Mann-Whitney *U* tests. Benjamini-Hochberg False Discovery Rate-adjusted p-values are shown. The effect size is presented as numbers below the p-values in each graph, representing fold change in factor concentration (OSCC patients versus Healthy group).

Our analysis revealed significantly elevated concentrations of seventeen factors in samples from OSCC patients, ranging from cytokines and chemokines associated with early-phase inflammation (IFN-α, IL-1α, IL-8 and CCL3), immune regulation (IL-1RA and IL-10), lymphocyte activation and recruitment (TNF-α, CXCL9, CXCL10, CCL27, IL-2, and IL-17A), and growth factors (β-NGF, FGF2, G-CSF, HGF and PDGFB) (**Figure 1C**).

To evaluate whether these soluble factor profiles could distinguish OSCC patients from healthy individuals, we performed unsupervised hierarchical clustering and principal component analysis (PCA) using only differentially expressed factors. Both approaches yielded largely distinct groupings, with partial overlap among individual samples (**Figures 2A, B**), indicating a robust and distinct systemic immune signature associated with OSCC.

**Figure 2:**
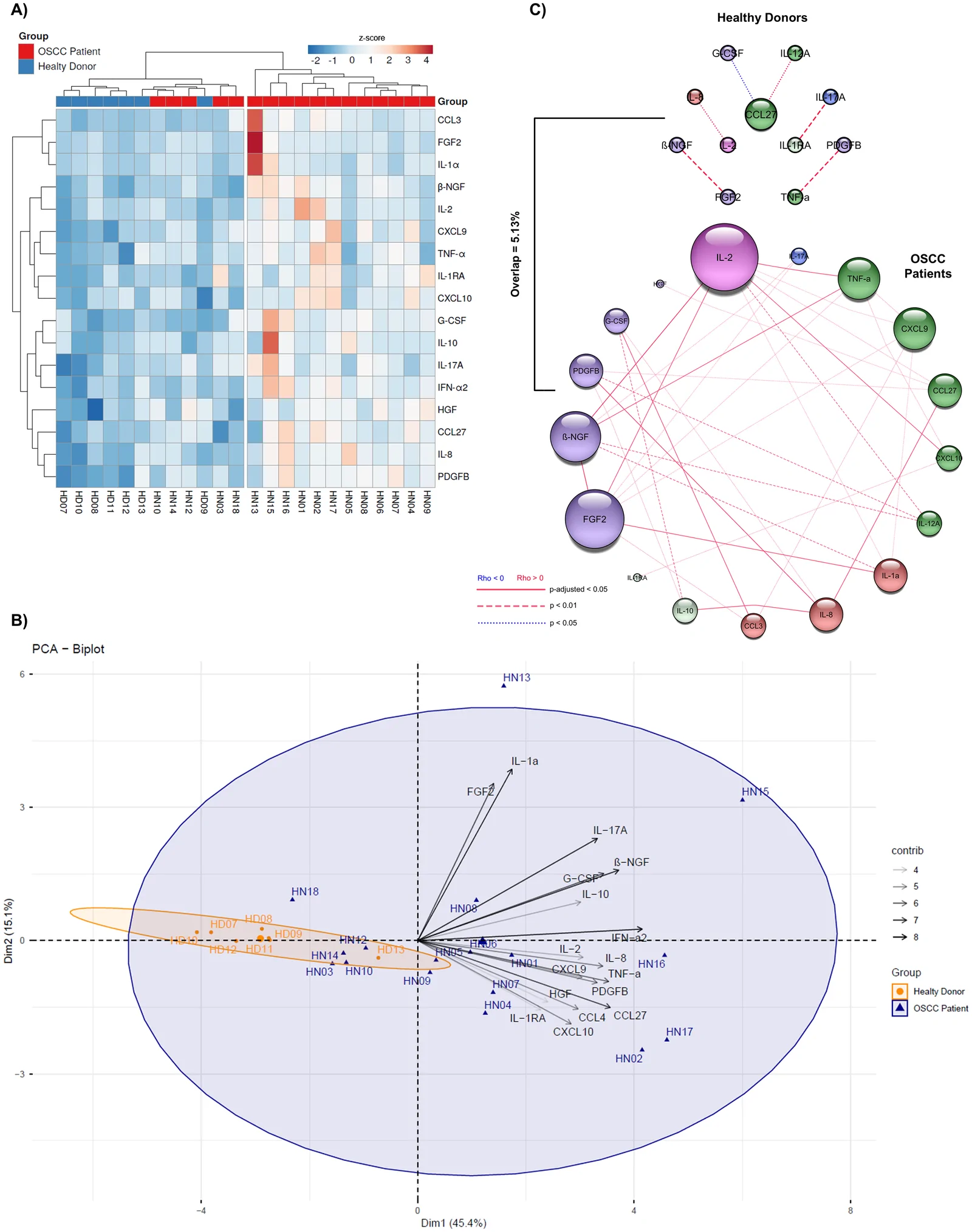
Systemic immune mediators segregate OSCC from controls and form highly connected networks. Patients with stage III or IV oral squamous cell carcinoma (OSCC) were recruited and had blood drawn before the administration of any kind of treatment. Blood from healthy donors was used as a control. Plasma analytes with significant differences (adjusted p-value <0.05) between healthy (n = 7) and OSCC patients (n = 17), as shown in **Figure 1**, were chosen for further analysis. All data were normalized by Ln(x+1) prior to cluster analysis. Raw data were used for multiple Spearman’s correlation analysis. **(A)** Heatmap of seventeen differentially expressed immune factors, constructed using Euclidean distance and Ward Linkage. **(B)** Principal Component Analysis biplot graph of seventeen differentially expressed immune factors depicting the patient (OSCC Patients; n = 17) and control (Healthy Donors; n = 7) groups, as well as each factor. Arrows indicate the contribution associated with dimension 1 (X axis) and dimension 2 (Y axis). **(C)** Correlation networks represent significant (p-value < 0.05) interactions between the selected factors. P-values were further segregated and represented as distinct edge types. Red connector lines represent correlations with positive coefficients and blue connector lines represent those with negative coefficients. Node diameter is proportional to the number of correlations each factor makes. Factors were grouped and color-coded based on similar characteristics or functions. “Overlap” represents the percentage of identical interactions shared between the groups.

PCA revealed that some of the top contributing variables along both principal dimensions were factors involved in cell proliferation and tissue remodeling, including β-NGF, FGF2, G-CSF, PDGFB, HGF and IL-2. This proliferative signature was also evident in correlation network analysis, which showed complex and highly interconnected relationships among all seventeen upregulated immune factors in OSCC patients (**Figure 2C**; **Supplementary Figure 3A**). In contrast, healthy individuals exhibited fewer overall interactions and a disconnected network.

Notably, nearly all the OSCC-specific correlations involved factors associated with lymphocyte proliferation and stromal remodeling (**Supplementary Figure 3B**). When mediators were grouped by function, the OSCC plasma profile emerged as a mixed proinflammatory and regulatory network, dominated by interactions involving lymphocyte proliferation, tissue remodeling, and T_H_1-related responses (**Supplementary Figure 3C**).

### Compartment-specific differences in immune cell frequencies in OSCC patients

Given the distinct systemic soluble immune profiles observed between OSCC patients and healthy donors, we next focused on characterizing the cellular immune composition within OSCC patients across different tissue compartments, namely, blood, peritumor and tumor. Using multiparametric flow cytometry, we assessed the frequencies of diverse immune cell subsets, including lymphoid and myeloid populations, regulatory T cells, mucosal-associated invariant T (MAIT-like) cells and cells expressing exhaustion-, memory- or activation-associated markers (**Supplementary Table 1**).

To explore the compartmental distribution of these cell populations, we first applied the UMAP algorithm to visualize clustering patterns based on flow cytometry data (**Supplementary Figure 4**). This revealed clear separation of immune cell populations based on their tissue of origin, as indicated by black arrows. These differing patterns were observed in both our myeloid and lymphoid cytometry panels (**Supplementary Figures 4A, B** – right panels). In parallel, PhenoGraph clustering identified distinct cellular subsets with variable distribution across compartments (**Supplementary Figure 4A, B** – left panels), suggesting tissue-specific enrichment of certain cell types.

Because unsupervised clustering algorithms can be influenced by low-abundance populations for a limited number of individuals, we performed a targeted comparative analysis of specific immune subsets across compartments (**Figure 3**). To do so, we performed a paired analysis based on Friedman’s test and Dunn’s *post-hoc* and further evaluated population frequency across distinct compartments with an ordinal trend analysis (Page’s L tests). Aside from the frequency of live cells (data not shown), the median frequencies of several cell populations differed significantly among blood, peritumor, and tumor samples. Specifically, in blood we observed higher frequencies of immune cells expressing cytotoxic markers, including Granzyme B^+^CD161^+^ NK cells, activated HLA-DR^+^ B cells and single-positive CD95 or PD-1 cells, in addition to CD4^-^ CD8^-^ CD3^+^ MAIT-like cells (TCR Vα7.2^+^CD161^+^) and CD161^+^ cells (**Figure 3A**). The peritumor area was characterized by CD4^+^ MAIT-like cells and CD161^+^ cells, effector memory cells, activated cells, regulatory T cells and cells expressing PD-L1 (**Figure 3B**). Tumor samples were enriched for CD25^+^FoxP3^+^ T cells, CD95^+^PD-1^+^ lymphocytes and PD-L1^+^ myeloid cells (**Figure 3C**). Interestingly, the ordinal trend analysis indicated a progressive enrichment of PD-1^+^CD95^+^, CD25^+^FoxP3^+^ and PD-L1^+^ cells along the blood-peritumor-tumor axis (**Supplementary Table 4**). We highlighted cell subsets with a significant trend (adjusted p-value below 0.05 in Page’s L tests) and an R² above 0.8 in Line Trend analysis with a star next to the population’s name (**Figure 3**).

**Figure 3:**
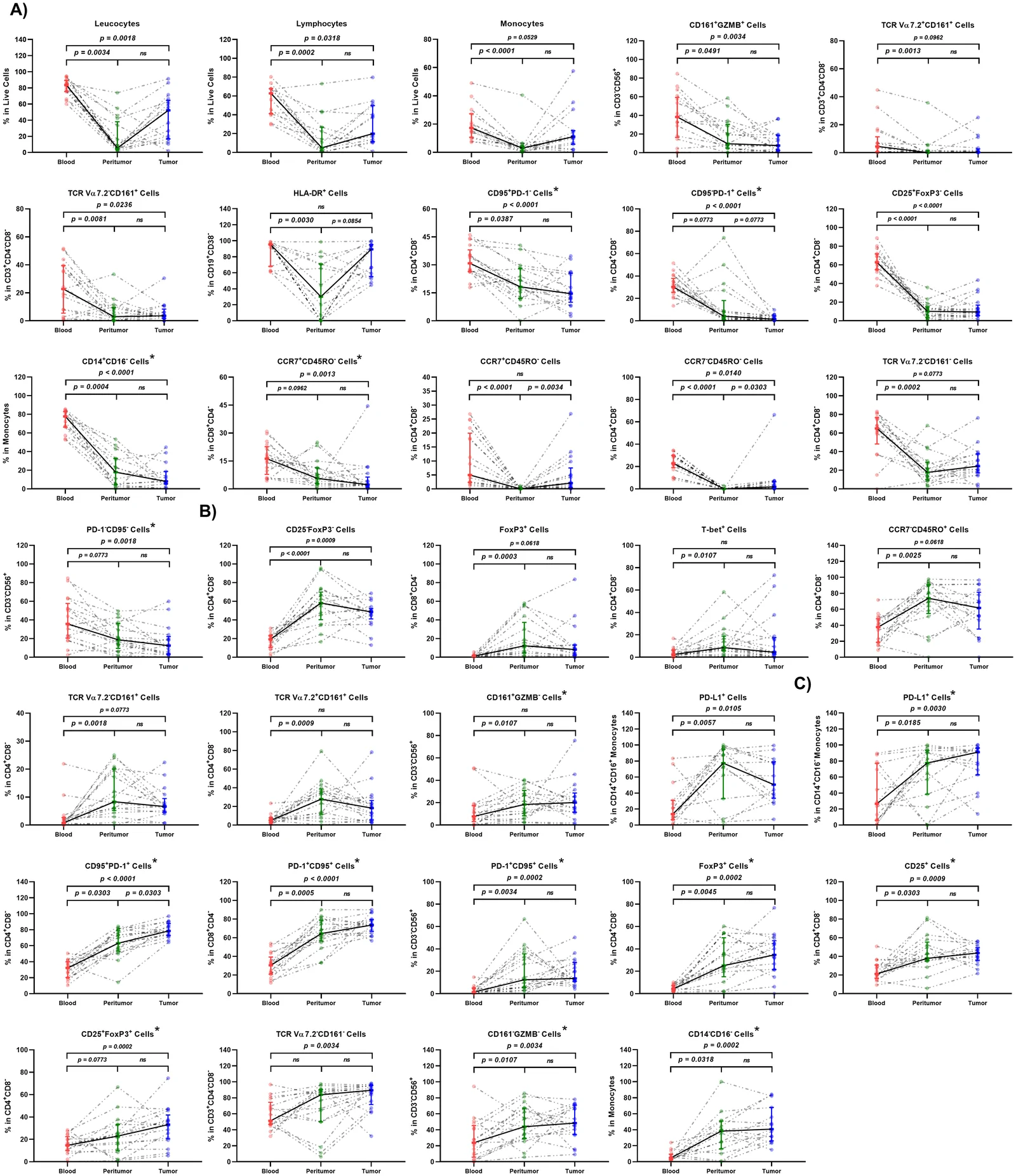
Paired analysis highlights compartment-specific cell distributions. Patients with stage III or IV oral squamous cell carcinoma (OSCC) were recruited and had blood drawn before the administration of any kind of treatment. Peripheral Blood Mononuclear Cells (PBMC) were obtained through density gradient centrifugation and assessed for immune marker expression by flow cytometry. Cells from tumor and peritumor were obtained through a dissociation protocol. Cytometry staining was done on the same day the samples were collected. **(A–C)** Graphs show the frequency of distinct leucocyte cell populations measured across three compartments in paired samples of OSCC patients. **(A)** Populations with higher frequency in blood samples. **(B)** Populations with higher frequency in peritumor samples. **(C)** Populations with higher frequency in tumor samples. Comparisons were performed using Friedman test with Dunn’s *post-hoc* test. Matched samples were available from 17 patients for Panel 1 populations and 15 patients for Panel 2 populations, as detailed in **Figure 1B**. Graphs show median frequencies (% of parent population) and interquartile ranges. Benjamini-Hochberg False Discovery Rate-adjusted p-values are shown. Population names are shown above each graph, and the y-axis indicates the parent population. Stars identify populations with an adjusted p-value below 0.05 in Page’s L trend analysis and R² above 0.8 in line-trend analysis (**Supplementary Table 4**).

To complement this analysis, we repeated the comparison using the full cohort and evaluating up to 24 blood, 18 tumor and 17 peritumor samples (**Supplementary Figure 5**). The direction and significance of the compartmental differences were largely preserved: populations enriched in blood included Granzyme B^+^CD161^+^ NK cells, CD4^-^ CD8^-^ CD3^+^ TCR Vα7.2^-^CD161^+^ cells and PD-1^+^ or CD95^+^ single-positive cells. Conversely, CD95^+^PD-1^+^ T and NK cells, CD25^+^FoxP3^+^ cells, and PD-L1^+^ myeloid cells were enriched in tumor samples. Because the unpaired analysis (**Supplementary Figure 5**) retains all available samples, including patients with incomplete sampling, and uses healthy-donor PBMC as a reference, whereas the paired analysis (**Figure 3**) maximizes within-patient comparability, the agreement between the two approaches reinforces the compartment-specific immune remodeling described above.

### Unsupervised cluster analysis highlights distinct immune landscapes in OSCC compartments

To explore whether immune profiles from distinct tissue compartments segregate without supervision, we applied principal component analysis (PCA) and hierarchical clustering (**Figure 4**). To avoid data imputation, the following cluster and cytometry analyses were done with the fourteen samples that had full evaluation of both cytometry panels across all compartments. Interestingly, the cluster analysis resulted in a heatmap without aggregation based on clinical data such as tumor site, clinical stage or lymph node metastasis. However, a strong compartmental distinction was revealed with blood samples forming a coherent and distinct cluster, while tumor and peritumor samples showed partial overlap, suggesting some shared immune features within the tumor microenvironment (**Figure 4A**). The PCA analysis further reinforced these findings. Samples from each compartment were plotted in reduced-dimensional space and grouped into partially or non-overlapping ellipses. Blood samples clustered distinctly along dimension 1 (accounting for 35.7% of the variance), while tumor and peritumor samples were more closely aligned along dimension 2 (15.4% of the variance) (**Figure 4B**). Immune cell subsets expressing activation markers — including NK cells, CD4^+^ T cells and classical monocytes — were associated with dimension 1 and aligned with the blood cluster. In contrast, CD95^+^PD-1^+^ T and NK cells, CD25^+^FoxP3^+^ T cells, and CD4^+^ MAIT-like cells contributed heavily to dimension 2 and were enriched in tumor and peritumor samples (**Figure 4B**). Interestingly, populations such as CD4^+^ MAIT-like cells and CD95^+^PD-1^+^ CD4^+^ and CD8^+^ T cells contributed most to the variance in the regions where peritumor and tumor samples clustered (**Figure 4C**). Regarding the blood compartment, overall leucocytes contributed most to the variance, with cells expressing CD161, Granzyme B and PD-1 or CD95 alone being important contributors (**Figure 4C**).

**Figure 4:**
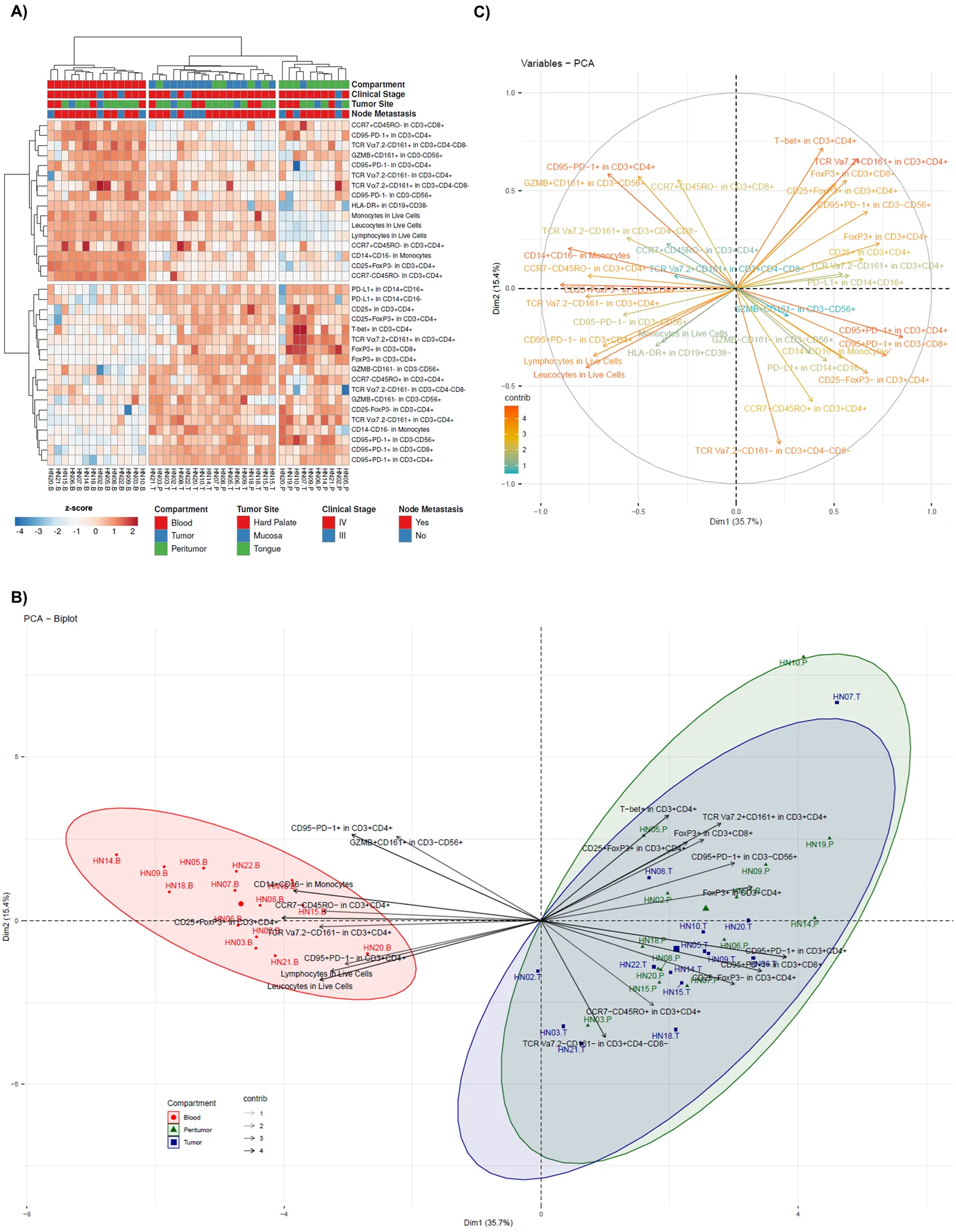
Unsupervised analyses reveal compartment-specific immune patterns across blood, peritumor and tumor. Patients with stage III or IV oral squamous cell carcinoma (OSCC) were recruited and had blood drawn before the administration of any kind of treatment. Peripheral Blood Mononuclear Cells (PBMC) were obtained through density gradient centrifugation and assessed for immune marker expression by flow cytometry. All cell populations with significant differences identified in **Figure 3** (adjusted p-value < 0.05) were used as a basis for unsupervised cluster analysis and principal component analysis. To avoid data imputation, we selected only patients with full assessment of both cytometry panels for each compartment, resulting in 14 samples. All data were normalized by Ln(x+1) prior to cluster analysis. **(A)** Heatmap of differentially expressed immune populations, constructed using Euclidean distance and Ward linkage. **(B)** Principal Component Analysis biplot graph of differentially expressed immune populations depicting different compartments (blood, tumor and peritumor), as well as the top 20 populations which presented the highest variance. Arrows indicate the contribution associated with dimension 1 (x axis) and dimension 2 (y axis). **(C)** Variables graph depicting all factors and their contribution to variance in a color scale.

### Correlation analysis reveals immune profile shifts across tissue compartments

To gain further insight into how immune cell behavior is shaped by tissue localization, we performed multiple Spearman correlation analyses, integrating cellular and soluble immune data. Specifically, we examined correlations between cell populations and soluble mediators that were differentially abundant across compartments and soluble factors from our panel, considering an FDR-adjusted p-value below 0.05. Factors were color-coded by functional category and ranked by the total number of observed interactions (**Figure 5**; **Supplementary Figure 6**).

**Figure 5:**
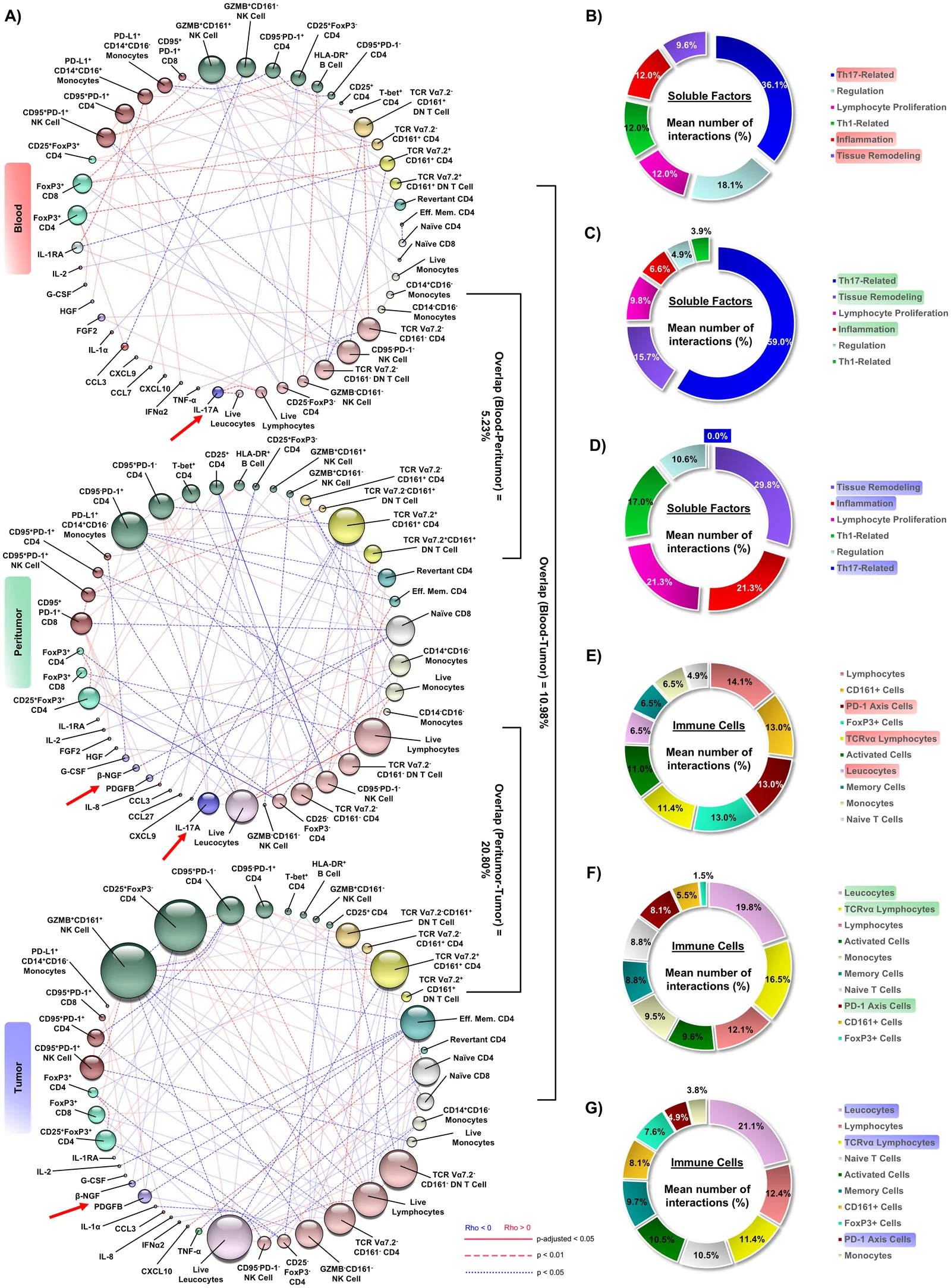
Distinct compartmental correlation networks involve activation-associated, PD-1-axis markers, and MAIT-like populations. Patients with stage III or IV oral squamous cell carcinoma (OSCC) were recruited and had blood drawn by surgery day, before the administration of any kind of treatment. Peripheral Blood Mononuclear Cells (PBMC) were obtained through density gradient centrifugation and assessed for immune marker expression by flow cytometry. Differentially expressed factors (considering all soluble immune factors and differentially expressed cell populations identified in **Figures 1**, **3**) were selected for multiple Spearman’s correlation analysis. **(A)** Correlation networks representing significant (p-value < 0.05) correlations between the selected factors, considering the compartments blood, tumor, and peritumor (n = 14). Sphere diameter is directly associated with the number of correlations each factor makes. Red connector lines represent correlations with positive coefficients and blue connector lines represent those with negative coefficients. P-values were further segregated and represented as distinct edge types. Factors were grouped and color-coded based on similar characteristics or functions. “Overlap” represents the percentage of identical interactions shared between the groups. Correlations made between soluble factors were removed for clarity. **(B–D)** Mean number of interactions of each group of soluble factors (segregated by function as detailed in **Supplementary Table 3**) considering the compartments blood **(B)**, peritumor **(C)** and tumor **(D)**. **(E–G)** Mean number of interactions of each group of cell populations (segregated by function as detailed in **Supplementary Table 3**) considering the compartments blood **(E)**, peritumor **(F)** and tumor **(G)**. Factors are presented in decreasing order of proportional interactions.

The analysis resulted in three distinct correlation networks and matrices. Correlation networks revealed low similarities across compartments, with tumor and peritumor sharing about 21% of all interactions and blood sharing about 5% of interactions with peritumor and 11% with tumor (**Figure 5A**). Because all soluble factors were measured in plasma, the differences across networks reflect distinct cell-frequency correlates of the same systemic mediators rather than compartment-specific cytokine levels, as highlighted by red arrows (**Figure 5A**). For example, IL-17A (measured in plasma) correlated with blood and peritumor cell frequencies but showed no significant correlations with tumor cell frequencies, consistent with a gradient of immune states across compartments. Conversely, the growth factors PDGFB and β-NGF presented interactions only among peritumor and tumor samples (**Figure 5A**). Further breakdown by cytokine class revealed that T_H_17-related factors dominated correlations in both blood and peritumor, whereas the opposite pattern was observed in tumor samples (**Figures 5B–D**). Interestingly, factors related to tissue remodeling and inflammation, accounting for half of all soluble factor correlations in tumor, having less prominence in peritumor and blood. (**Figures 5B–D**). This shift was mirrored in cellular correlation patterns: cells expressing TCR Vα7.2 had a high proportion of interactions in all compartments, especially in peritumor and tumor (**Figures 5E–G**). In contrast, correlations involving overall leucocytes were enriched in both tumor and peritumor samples and were much less common in blood samples (**Figures 5F, G**).

To investigate whether cell-frequency relationships across compartments are consistent with phenotypic differences among blood, peritumor and tumor regions, we conducted a second correlation analysis, this time linking frequencies of the same immune cell types across compartments (**Figure 6**; **Supplementary Figure 7**). For visual clarity, intra-compartment correlations (e.g., blood-to-blood) were excluded. Networks were color-coded by immune cell function and shaded by compartment (**Supplementary Table 3**).

**Figure 6:**
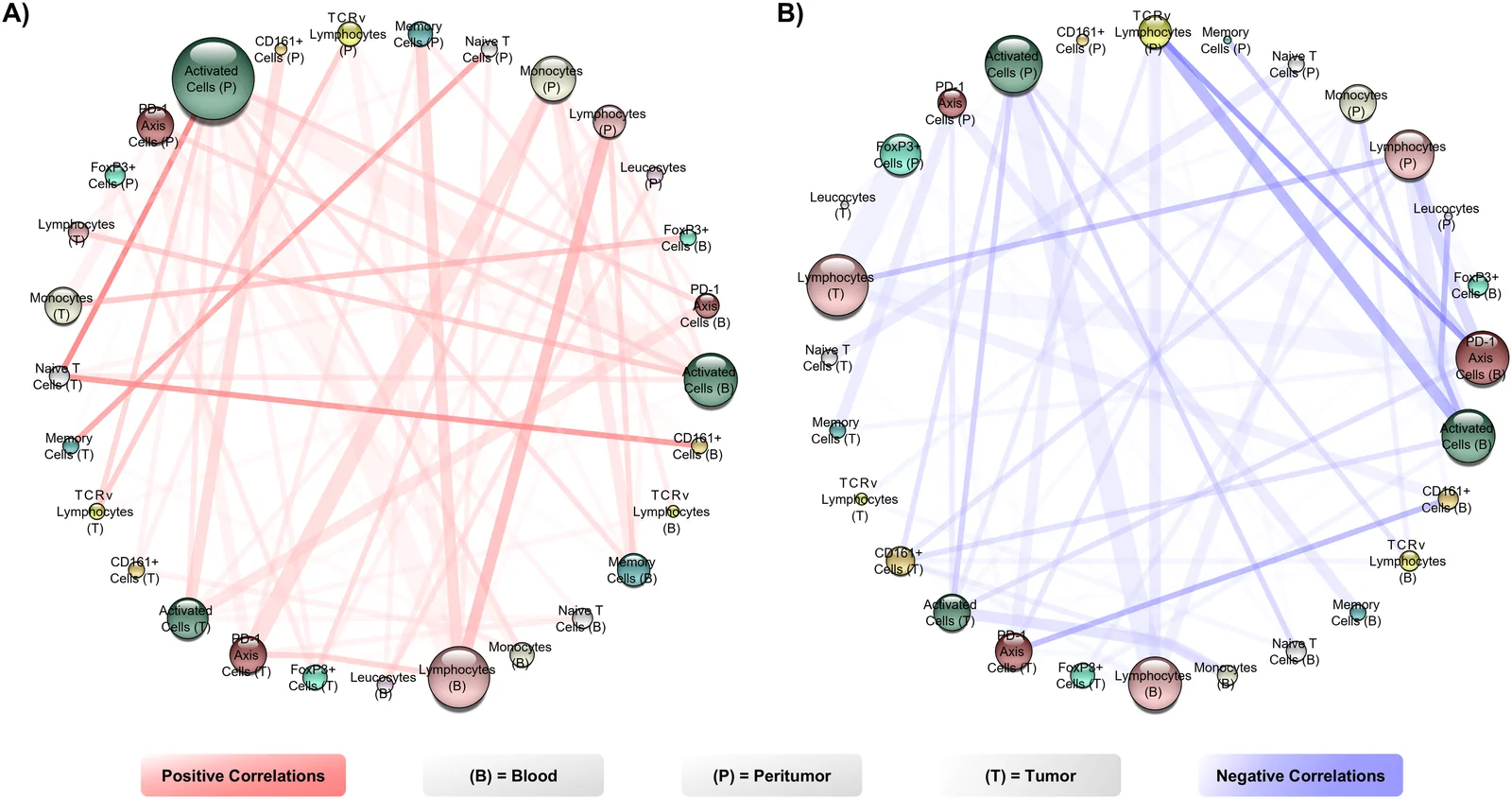
Cross-compartment correlations reveal compartment-specific immune relationships across blood, peritumor and tumor. Patients with stage III or IV oral squamous cell carcinoma (OSCC) were recruited and had blood drawn before the administration of any kind of treatment. Peripheral Blood Mononuclear Cells (PBMC) were obtained through density gradient centrifugation and assessed for immune marker expression by flow cytometry. Tumor and peritumor cells were obtained through a tissue dissociation protocol. Differentially expressed cell populations as identified in **Figure 3** (p-value < 0.05) were selected for multiple Spearman’s correlation analysis. The correlation networks represent significant (p-value < 0.05) correlations between the selected factors, considering the compartments blood, tumor and peritumor (n = 14). **(A)** Positive correlations are identified with red connector lines; stronger colors indicate higher correlation coefficients (range 0.5429 to 0.7647). **(B)** Negative correlations are identified with blue connector lines; stronger colors indicate lower correlation coefficients (range -0.5385 to -0.8348). All interactions between cells from the same compartment (e.g., blood-blood) were suppressed. Node diameter is proportional to the number of correlations the group of factors make, and line width correlates with the number of interactions made with another group. Cell populations were grouped and color-coded based on similar characteristics or functions.

This analysis revealed possible shifts in marker expression and population enrichment across compartments (**Figure 6**). For example, populations of activated cells in blood and peritumor correlated with one another, while myeloid and activated cells from peritumor correlated with the frequency of PD-1 axis cells (which include PD-1^+^CD95^+^ and PD-L1^+^ populations) in tumor samples (**Figure 6A**). Moreover, we observed that the frequency of PD-1 axis cells in the blood was inversely correlated particularly with populations of memory cells and TCR Vα7.2^+^ cells present in the peritumor (**Figure 6B**).

## Discussion

The tumor microenvironment (TME) in OSCC is shaped by reciprocal interactions among malignant, stromal, and immune cells and soluble mediators, with effects that may extend systemically (12). Although inhibitors of the PD-1/PD-L1 pathway have reshaped the management of recurrent or metastatic head and neck squamous cell carcinoma (20), their benefit remains limited to a subset of patients, motivating efforts to refine indications and enhance therapeutic efficacy in OSCC (21). Our study identifies distinct systemic, peritumor, and tumor-associated immune patterns in advanced, treatment-naive OSCC. The data supports compartment-specific differences and selected monotonic trends.

At the systemic level, OSCC patients showed a mixed immune signature characterized by increased concentrations of growth factors, cytokines and chemokines with pro-inflammatory and immunoregulatory roles. Previous studies reported systemic alterations in circulating analytes, including higher levels of pro-inflammatory cytokines (IL-1β, IL-6, IL-8, TNF-α), anti-inflammatory cytokines such as IL-10, chemokines (CXCL10, CCL2, CCL3), and angiogenic growth factors (VEGF, FGF2) (22, 23), together with reductions in IFN-γ, CCL4, and IL-13 (22). A larger plasma study (210 OSCC, 50 controls) further showed that a model based on IL-2, IL-1Ra, and MIF discriminated OSCC from controls with high sensitivity and specificity (24). In our preoperative plasma analysis, 17 analytes were elevated compared with healthy individuals, including cytokine IL-2 and the growth factors β-NGF, FGF2, G-CSF, HGF, and PDGFB, consistent with pathways related to lymphocyte activation and tissue remodeling. The convergence with prior reports supports these mediators as hypothesis-generating candidates for future validation (22–24).

Across cellular compartments, the paired analysis revealed distinct distributions rather than a uniformly intermediate peritumor phenotype. Peripheral blood showed higher frequencies of Granzyme B^+^CD161^+^ NK cells, HLA-DR^+^ B cells, single-positive PD-1 or CD95 cells, and CD4^-^CD8^-^CD3^+^ TCR Vα7.2^+^CD161^+^ MAIT-like cells, whereas tumor samples were enriched for CD25^+^FoxP3^+^ T cells, CD95^+^PD-1^+^ lymphocytes, and PD-L1^+^ myeloid cells. The peritumor compartment was characterized by CD4^+^ MAIT-like and CD161^+^ cells, effector-memory and activated populations, regulatory T cells, and PD-L1-expressing cells. Importantly, the ordinal trend analysis identified progressive enrichment of selected PD-1^+^CD95^+^, CD25^+^FoxP3^+^, and PD-L1^+^ populations along the blood-peritumor-tumor axis. Thus, monotonic trends were supported for specific populations, but not for the peritumor compartment as a whole. Because these phenotypes are marker-defined, they should not be interpreted as direct functional evidence of exhaustion or immunosuppression. Nevertheless, prior studies of MAIT cells in cancer (25),​ including associations between higher MAIT frequencies and improved responses to anti-PD-1 therapy, with more cytotoxic MAIT profiles in responders (26), provide a rationale for functional validation of the MAIT-like populations observed here.

Although features entering the correlation analyses were restricted to those that remained significant after FDR correction in the preceding group comparisons, the resulting networks should be interpreted as exploratory association maps. Nevertheless, we observed that IL-17A represented the largest share of soluble-factor correlations in blood and peritumor tissue, whereas tissue-remodeling and inflammatory factors were relatively more prominent in tumor. IL-17A correlated with cell frequencies in blood and peritumor but not in tumor, while PDGFB and β-NGF showed correlations only in the tissue networks. Because soluble mediators were measured in plasma, these associations do not represent local cytokine production, instead suggesting that the same circulating mediators relate differently to cell frequencies across compartments. The balance among T_H_1, T_H_2, and T_H_17 programs in cancer is strongly context-dependent and may vary with tumor type, anatomic site, and disease stage (27). This compartment-specific pattern is compatible with the context-dependent behavior of helper and stromal programs described in cancer and OSCC (28)​, but mechanistic direction cannot be inferred from these cross-sectional correlations.

Network overlaps further argue against a simple linear transitional model. Tumor and peritumor networks shared about 21% of interactions, compared with approximately 5% between blood and peritumor and 11% between blood and tumor. Thus, the two tissue compartments were more alike in network structure than either was to blood. However, the exact distance and orientation of peritumor sampling were not recorded, samples were dissociated before cytometry, and histological composition and sampling depth varied by anatomic site. Accordingly, these data cannot establish a spatial transition zone or determine whether immune remodeling begins outside the tumor core.

Taken together, our cross-compartment analyses indicate that immune activation in peripheral blood coexists with exhaustion-associated programs at the tumor site, while the peritumor compartment displays a distinct immune profile that partially overlaps with both systemic and tumor-associated features. Correlations across compartments further suggest that related immune populations may adopt different phenotypic states depending on tissue localization. Notably, peritumor and tumor networks shared more interactions with each other than either did with blood, supporting a closer immunological relationship between the two tissue compartments without establishing a spatially progressive transition. These findings may be consistent with broader field effects in oral carcinogenesis, including inflammatory and immunoregulatory changes described in oral potentially malignant disorders (OPMDs) (29–32). However, because OPMDs were not identified in our cohort and the exact spatial relationship between peritumor samples and the invasive tumor front was not recorded, these mechanisms remain hypothetical and require dedicated spatial and histopathological validation.

Our study is limited by a cross-sectional design, modest sample size, lack of healthy non-neoplastic oral mucosal controls and a limited number of control samples, and pre-analytical differences between PBMC isolation and tissue dissociation. These factors may affect cell recovery, viability, and marker expression, hindering our ability to clearly segregate tumor-specific effects from normal blood-versus-tissue compartmentalization. Furthermore, due to the nature of the disease and the goal of preserving functional tissue from the patients, the peritumor specimens were not sampled at a recorded distance or standardized orientation, while the tumor samples were not systematically collected from comparable depths. The distinct nature of the compartments (namely blood and solid tissue) also prevented a comparable cell count based on tissue weight. Nonetheless, the use of paired, multi-compartment profiling and integrated soluble and cellular readouts provides a coherent view of OSCC immunity that is hypothesis-generating. Specifically, the identification of plasma immune mediators and compartment-specific cellular patterns should therefore be considered exploratory signals for future spatial, functional, longitudinal, and clinical validation.

## Conclusion

In advanced OSCC, preoperative plasma and matched cellular profiling identified distinct systemic and tissue-associated immune patterns. Seventeen plasma mediators were increased relative to healthy donors, while paired cellular analyses identified compartment-specific distributions and significant ordinal trends for selected PD-1^+^CD95^+^, CD25^+^FoxP3^+^, and PD-L1^+^ populations. Peritumor and tumor networks shared more interactions with each other than with blood, but the current design does not establish a spatial transition zone or tumor-induced immune reprogramming. These findings are exploratory and provide hypotheses for future spatial, functional, longitudinal, and clinical validation studies.

## Data Availability

The raw data supporting the conclusions of this article will be made available by the authors, without undue reservation.
