# Supplementary material for: Systemic immune activation coexists with compartment-specific regulatory enrichment in matched blood, peritumor, and tumor tissue from advanced oral squamous cell carcinoma
Source: Front Immunol. 2026 Oct 1;17:1923358. doi: 10.3389/fimmu.2026.1923358 (PMC13669159; doi:10.3389/fimmu.2026.1923358)
Supplement: Supplementary file 1 [file SupplementaryFile1.pdf]

Supplementary Material

Supplementary Table 1. List of antibodies used for multiparametric flow cytometry.

PANEL 1: T LYMPHOCYTES AND NK CELLS

	Catalog #	Marker	Fluorochrome	Clone
**1**	612891	CD45	BUV805	HI30
**2**	564805	CD8	BUV496	RPA-T8
**3**	612918	CD25	BUV563	2A3
**4**	565065	CD3	BUV661	UCHT1
**5**	566355	CD4	BV750	SK3
**6**	657707	TCRγδ	APC-R700	11F2
**7**	561137	CD45RO	APC-H7	UCHL1
**8**	561636	CD95	PE-Cy7	DX2
**9**	566756	CCR7 (CD197)	BV650	2-L1-A
**10**	612791	PD-1 (CD279)	BUV737	EH12.1
**11**	749488	TCR Vα7.2	BV786	0F-5A12
**12**	566756	CD127	BB700	HILL7RM21
**13**	555518	CD56	APC	B159
**14**	561138	CD161	PE-Cy5	DX12
**15**	560047*	FoxP3	AlexaFluor 488	259D/C7
**16**	569089*	T-bet	PE-CF594	04-46
**17**	561142*	Granzyme B	BV510	GB11

PANEL 2: B CELLS AND MYELOID CELLS

	Catalog #	Marker	Fluorochrome	Clone
**1**	612891	CD45	BUV805	HI30
**2**	557758	CD16	APC-Cy7	3G8
**3**	560584	CD11c	PerCP-Cy5.5	HL3
**4**	560677	CD38	PE-Cy7	HIT2
**5**	569704	CD11b	BV650	ICRF44
**6**	568320	PD-L1	BV421	29E.2A3
**7**	565188	PD-L2	APC-R700	MIH1
**8**	555397	CD14	FITC	M5E2
**9**	561741	CD19	PE	HIB19
**10**	559866	HLA-DR	APC	G46-6

*Custom antibody, a similar can be found with the referred catalog number.

Supplementary Table 2. Flow cytometry event count summary.

Panel 1 (n = 25)	Total Count	Mean	Median	Lower Range	Higher Range
**Blood**	2398021	95921	90180	38702	168616
**Peritumor**	1902202	100116	40036	5208	498000
**Tumor**	2885130	151849	111311	18693	472000
**Healthy**	521204	65151	62046	57112	81247
**Panel 2 (n = 24)**	**Total Count**	**Mean**	**Median**	**Lower Range**	**Higher Range**
**Blood**	2590150	107923	114353	61635	173763
**Peritumor**	2203789	129635	41654	501	786000
**Tumor**	2850670	167686	147622	19292	704000
**Healthy**	595194	74399	67116	61496	109741

Supplementary Table 3. Nomenclature used for correlation analysis.

Cell Population or Soluble Factor	Nomenclature	Cell Population or Soluble Factor	Nomenclature
$CD25^+$ in $CD3^+CD4^+$	Activated Cells	$CCR7^+CD45RO^-$ in $CD3^+CD8^+$	Naïve T Cells
$CD95^+PD\text{-}1^-$ in $CD3^+CD4^+$	Activated Cells	$CCR7^-CD45RO^+$ in $CD3^+CD4^+$	Memory Cells/Eff. Mem. Cells
$CD95^-PD\text{-}1^+$ in $CD3^+CD4^+$	Activated Cells	$CCR7^+CD45RO^-$ in $CD3^+CD4^+$	Naïve T Cells
$T\text{-}bet^+$ in $CD3^+CD4^+$	Activated Cells	$CCR7^-CD45RO^-$ in $CD3^+CD4^+$	Memory Cells/Revertant CD4
$CD161^+GZMB^+$ in $CD3^-CD56^+$	Activated Cells	Monocytes in Live Cells	Monocytes
$HLA\text{-}DR^+$ in $CD19^+CD38^-$	Activated Cells	$CD14^+CD16^-$ in Monocytes	Monocytes
$CD25^+$FoxP3- in $CD3^+CD4^+$	Activated Cells	$CD14^-CD16^-$ in Monocytes	Monocytes
TCR $V\alpha 7.2^+CD161^+$ in $CD3^+CD4^+$	TCRvα Lymphocytes	G-CSF	Tissue Remodeling
TCR $V\alpha 7.2^+CD161^+$ in $CD3^+CD4^-CD8^-$	TCRvα Lymphocytes	IL-2	Lymphocyte Proliferation
TCR $V\alpha 7.2^-CD161^+$ in $CD3^+CD4^+$	$CD161^+$ Cells	CCL3	Inflammation
TCR $V\alpha 7.2^-CD161^+$ in $CD3^+CD4^-CD8^-$	$CD161^+$ Cells	IL-1RA	Regulation
$CD161^-GZMB^+$ in $CD3^-CD56^+$	Activated Cells	IL-10	Regulation
$CD25^+$FoxP3+ in $CD3^+CD4^+$	$FoxP3^+$ Cells	IL-17A	T_H17-Related
FoxP3+ in $CD3^+CD4^+$	$FoxP3^+$ Cells	IFNα2	T_H1-Related
FoxP3+ in $CD3^+CD8^+$	$FoxP3^+$ Cells	CCL27	T_H1-Related
$CD25^-$FoxP3- in $CD3^+CD4^+$	Lymphocytes	IL-1α	Inflammation
TCR $V\alpha 7.2^-CD161^-$ in $CD3^+CD4^+$	Lymphocytes	CXCL10	T_H1-Related

TCR Vα7.2$^-$CD161$^-$ in CD3$^+$CD4$^-$CD8$^-$	Lymphocytes	IL-8	Inflammation
CD161$^-$GZMB$^-$ in CD3$^-$CD56$^+$	Lymphocytes	CXCL9	T_H1-Related
CD95$^-$PD-1$^-$ in CD3$^-$CD56$^+$	Lymphocytes	TNF-α	T_H1-Related
Leucocytes in Live Cells	Lymphocytes	ß-NGF	Tissue Remodeling
CD95$^+$PD1$^+$ in CD3$^+$CD8$^+$	PD-1 Axis	FGF2	Tissue Remodeling
PD-1$^+$CD95$^+$ in CD3$^+$CD4$^+$	PD-1 Axis	HGF	Tissue Remodeling
CD95+PD-1+ in CD3-CD56+	PD-1 Axis	PDGFB	Tissue Remodeling
PD-L1$^+$ in CD14$^+$CD16$^+$	PD-1 Axis	Leucocytes in Live Cells	Leucocytes
PD-L1$^+$ in CD14$^+$CD16$^-$	PD-1 Axis	Obs.: CD3$^+$ TCR Vα7.2$^+$CD161$^+$ were deemed as "MAIT-like cells" in the text.	

Supplementary Table 4. Page's L Trend Test and Trend Line Analyses results summary.

	Median Frequency (%)			Line Trend Analysis		Page's L Test	
**Population**	**Blood**	**Peritumor**	**Tumor**	**Equation**	**R^2**	**p-value**	**BH**
Leucocytes in Live Cells	83.5	5.28	52.1	y = -15.7x + 78.36	0.1591	6.07E-03	9.64E-03
$CD95^{+}PD\text{-}1^{+}$ in $CD3^{+}CD8^{+}$	30.4	64.4	73.5	y = 21.55x + 13	0.8999	6.58E-07	8.88E-06
$FoxP3^{+}$ in $CD3^{+}CD8^{+}$	1.04	12.2	8.16	y = 3.56x + 0.0133	0.397	2.06E-02	2.78E-02
$CCR7^{+}CD45RO^{-}$ in $CD3^{+}CD8^{+}$	16.2	5.63	2.2	y = -7x + 22.01	0.9202	4.39E-04	1.08E-03
$CD25^{+}$ in $CD3^{+}CD4^{+}$	21.2	37.9	43.5	y = 11.15x + 11.9	0.9237	3.16E-04	8.54E-04
$CD95^{+}PD\text{-}1^{-}$ in $CD3^{+}CD4^{+}$	30.8	18.2	14.5	y = -8.15x + 37.467	0.9096	2.65E-05	1.43E-04
$CD95^{+}PD\text{-}1^{+}$ in $CD3^{+}CD4^{+}$	32.1	63	78.5	y = 23.2x + 11.467	0.9646	2.68E-07	7.22E-06
$CD95^{-}PD\text{-}1^{+}$ in $CD3^{+}CD4^{+}$	30.3	3.97	1.33	y = -14.485x + 40.837	0.8177	8.24E-06	7.41E-05
$CD25^{+}FoxP3^{+}$ in $CD3^{+}CD4^{+}$	14.6	22.9	33.1	y = 9.25x + 5.0333	0.9965	8.00E-05	2.70E-04
$CD25^{+}FoxP3^{-}$ in $CD3^{+}CD4^{+}$	62.6	10.1	9.3	y = -26.65x + 80.633	0.7613	2.65E-05	1.43E-04
$CD25^{-}FoxP3^{-}$ in $CD3^{+}CD4^{+}$	19.2	58.1	48.5	y = 14.65x + 12.633	0.5226	3.16E-04	8.54E-04
$CCR7^{-}CD45RO^{+}$ in $CD3^{+}CD4^{+}$	37.6	73.8	61.7	y = 12.05x + 33.6	0.4276	2.06E-02	2.78E-02
$CCR7^{+}CD45RO^{-}$ in $CD3^{+}CD4^{+}$	4.96	0	2.04	y = -1.46x + 5.2533	0.3430	1.45E-01	1.50E-01
$CCR7^{-}CD45RO^{-}$ in $CD3^{+}CD4^{+}$	22.8	0	1.81	y = -10.495x + 29.193	0.6858	4.66E-03	7.86E-03
TCR $V\alpha7.2^{-}CD161^{+}$ in $CD3^{+}CD4^{+}$	0.75	8.33	6.63	y = 2.94x - 0.6433	0.5464	2.58E-02	3.16E-02
TCR $V\alpha7.2^{+}CD161^{+}$ in $CD3^{+}CD4^{+}$	4.96	27.7	18.2	y = 6.62x + 3.7133	0.336	3.96E-02	4.45E-02
TCR $V\alpha7.2^{-}CD161^{-}$ in $CD3^{+}CD4^{+}$	64.9	17.6	24.4	y = -20.25x + 76.133	0.627	2.58E-02	3.16E-02
$T\text{-}bet^{+}$ in $CD3^{+}CD4^{+}$	2.34	8.57	4.35	y = 1.005x + 3.0767	0.0999	2.30E-01	2.30E-01
$FoxP3^{+}$ in $CD3^{+}CD4^{+}$	4.24	25	34.6	y = 15.18x - 9.08	0.9569	5.57E-05	2.51E-04
TCR $V\alpha7.2^{-}CD161^{+}$ in $CD3^{+}CD4^{-}CD8^{-}$	22.7	2.89	3.47	y = -9.615x + 28.917	0.7274	7.86E-03	1.18E-02
TCR $V\alpha7.2^{+}CD161^{+}$ in $CD3^{+}CD4^{-}CD8^{-}$	4.42	0	0.33	y = -2.045x + 5.6733	0.6898	3.21E-02	3.76E-02
TCR $V\alpha7.2^{-}CD161^{-}$ in $CD3^{+}CD4^{-}CD8^{-}$	51.1	83.8	89.4	y = 19.15x + 36.467	0.857	1.12E-03	2.02E-03
$CD161^{+}GZMB^{+}$ in $CD3^{-}CD56^{+}$	38.2	9.51	7.69	y = -15.255x + 48.977	0.7946	1.12E-03	2.02E-03
$CD161^{+}GZMB^{-}$ in $CD3^{-}CD56^{+}$	7.74	18.4	20	y = 6.13x + 3.12	0.846	8.63E-02	9.33E-02
$CD161^{-}GZMB^{-}$ in $CD3^{-}CD56^{+}$	23.7	43.8	48.4	y = 12.35x + 13.933	0.884	1.12E-03	2.02E-03
$CD95^{+}PD\text{-}1^{+}$ in $CD3^{-}CD56^{+}$	1.25	12.4	13.5	y = 6.125x - 3.2	0.8168	8.00E-05	2.70E-04
$CD95^{-}PD\text{-}1^{-}$ in $CD3^{-}CD56^{+}$	35.6	18.6	12.3	y = -11.65x + 45.467	0.9343	6.04E-04	1.36E-03
Lymphocytes in Live Cells	62.7	4.86	19.8	y = -21.45x + 72.02	0.5104	1.06E-02	1.48E-02
$HLA\text{-}DR^{+}$ in $CD19^{+}CD38^{-}$	95.4	30.0	89.3	y = -3.05x + 77.667	0.0071	2.73E-01	2.73E-01
Monocytes in Live Cells	17.2	2.95	11.0	y = -3.1x + 16.583	0.1882	1.76E-02	2.06E-02

PD-L1$^+$ in CD14$^+$CD16$^+$	13.6	77.3	50.5	y = 18.45x + 10.233	0.3328	3.49E-03	6.10E-03
CD14$^+$CD16$^-$ in Monocytes	77.7	17.8	7.9	y = -34.9x + 104.27	0.8539	1.18E-05	8.24E-05
PD-L1$^+$ in CD14$^+$CD16$^-$	26.5	77.5	91.0	y = 32.25x + 0.5	0.8987	1.02E-03	2.37E-03
CD14$^-$CD16$^-$ in Monocytes	4.76	38.2	40.6	y = 17.92x - 7.9867	0.8000	5.90E-05	2.07E-04

Equations and Coefficients (R^2) are related to a Trend Line made with each cell population's median frequency. This Line was constructed with a built-in Microsoft Excel tool (Microsoft 365). Page’s L Trend tests were used to assess decreasing or increasing trends in cell frequency across distinct compartments. BH = Benjamini-Hochberg False Discovery Rate p-value multiple hypotheses correction.

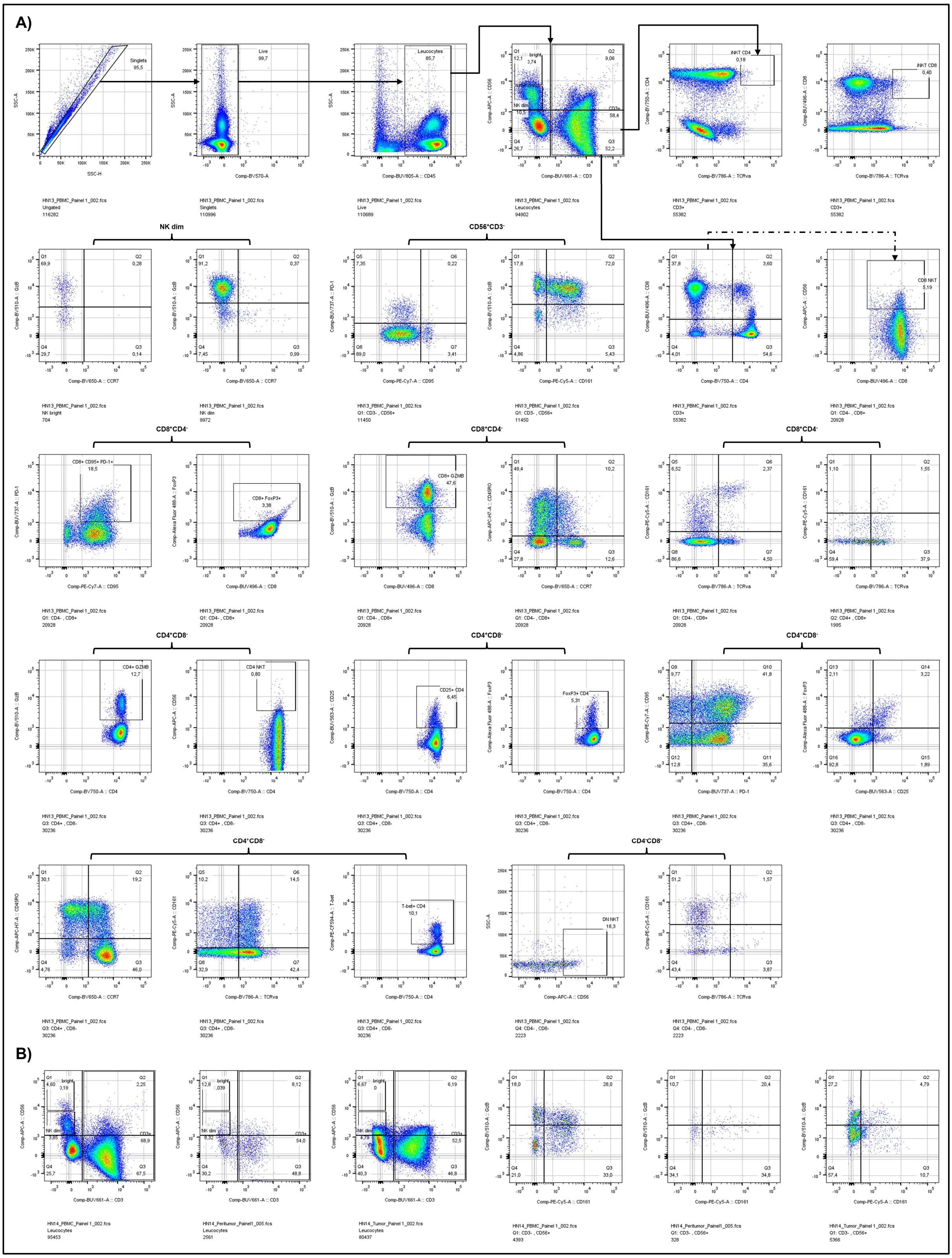

Supplementary Figure 1. Gating Strategy for Panel 1. Patients with stage III or IV oral squamous cell carcinoma (OSCC) were recruited and had blood drawn before the administration of any kind of treatment. Peripheral Blood Mononuclear Cells (PBMC) were obtained through density gradient centrifugation and assessed for immune marker expression by flow cytometry. Cells from tumor and peritumor were obtained through a dissociation

protocol. Cytometry staining was done on the same day the samples were collected. **(A)** Representation of gating strategy for main Lymphocyte populations assessed with Panel 1. **(B)** Representative plots of selected markers within a cell subset. Not all marker combinations are shown. Panel information can be found in **Supplementary Table 1**.

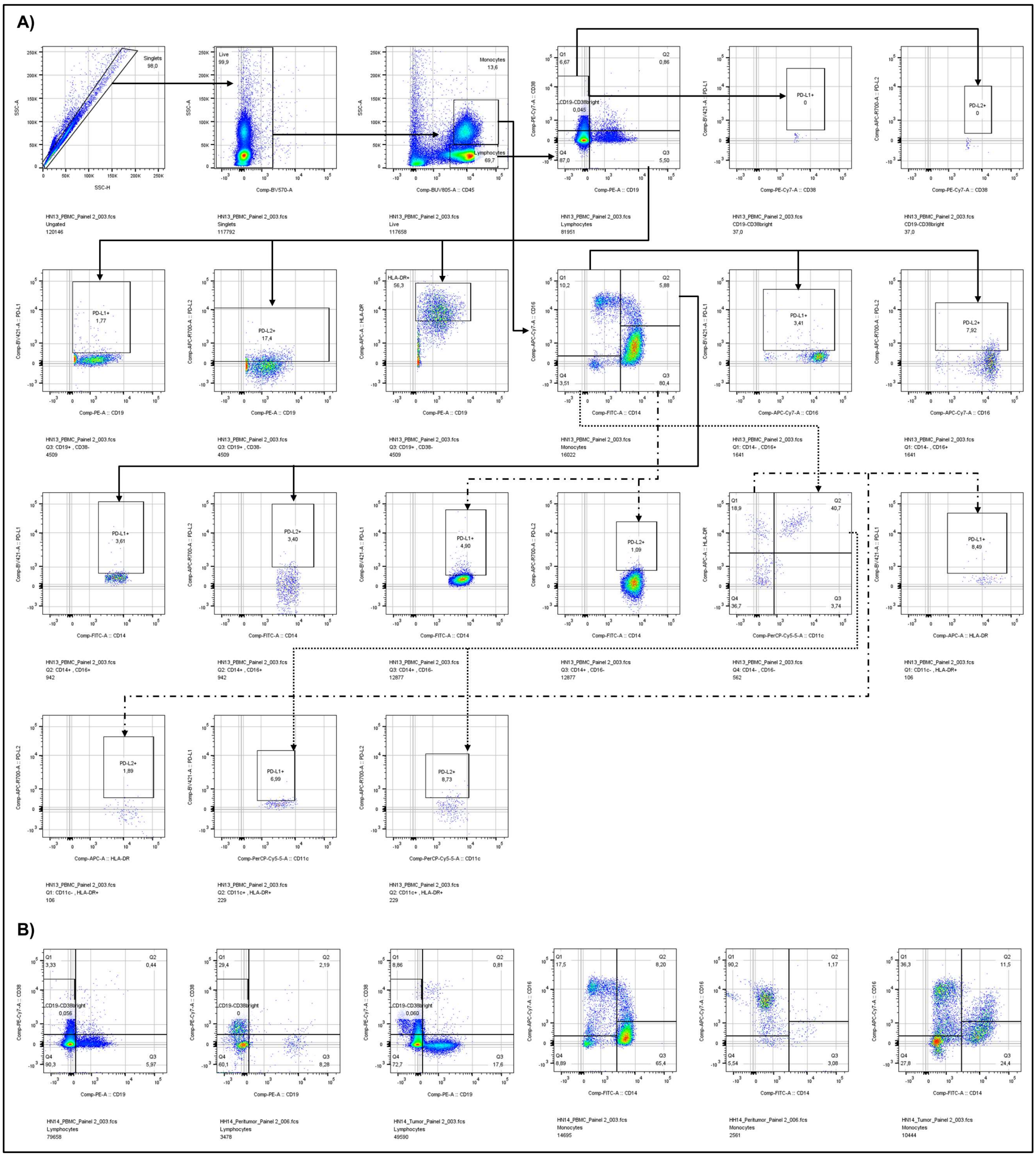

Supplementary Figure 2. Gating Strategy for Panel 2. Patients with stage III or IV oral squamous cell carcinoma (OSCC) were recruited and had blood drawn before the administration of any kind of treatment. Peripheral Blood Mononuclear Cells (PBMC) were obtained through density gradient centrifugation and assessed for immune marker expression by flow cytometry. Cells from tumor and peritumor were obtained through a dissociation protocol. Cytometry staining was done on the same day the samples were collected. **(A)** Representation of gating strategy for main Lymphocyte and Monocyte populations assessed with Panel 2. **(B)** Representative plots of selected markers within a cell subset. Not all marker combinations are shown. Panel information can be found in **Supplementary Table 1**.

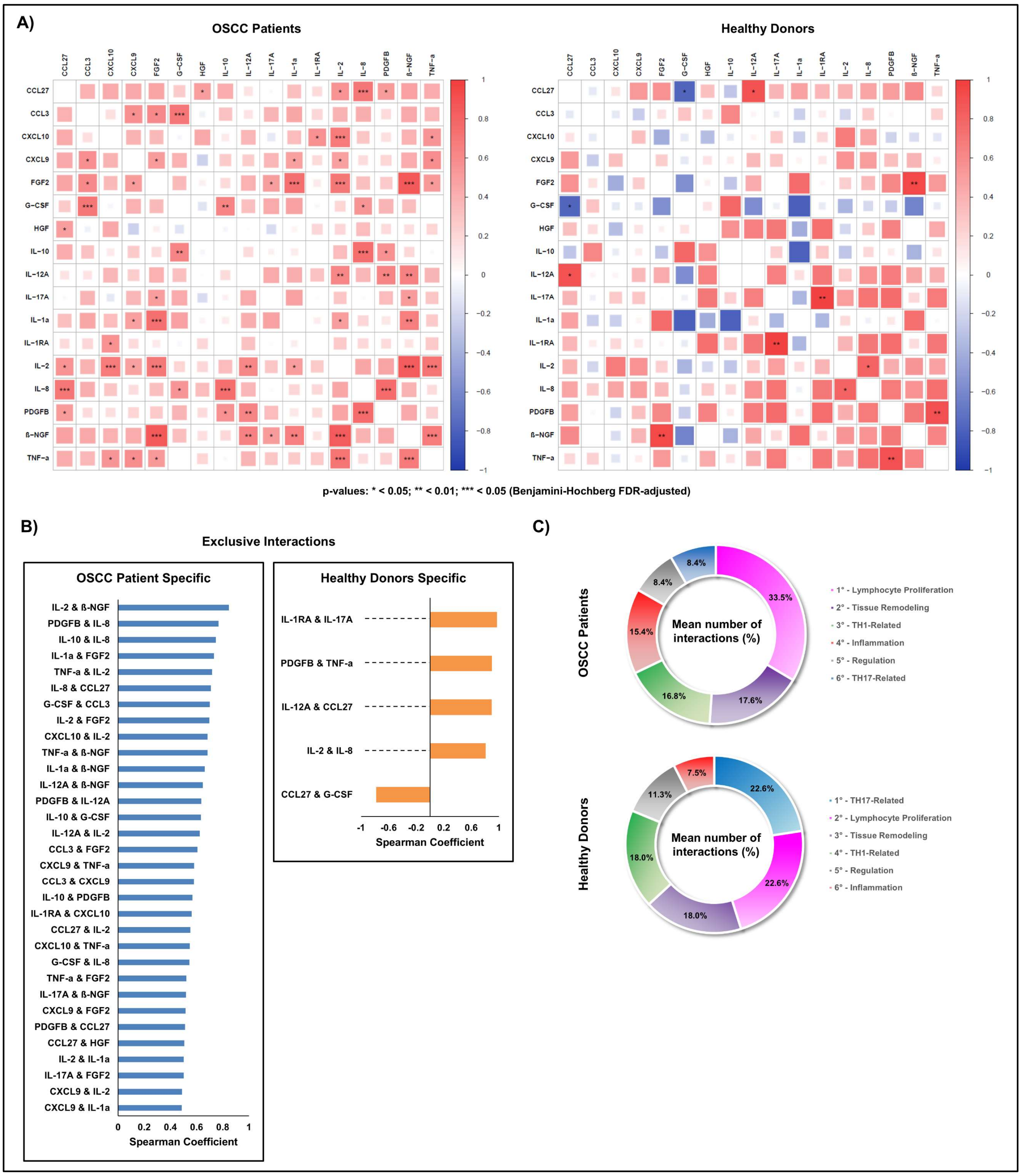

Supplementary Figure 3. Correlation analysis suggests an ongoing mixed immune response among oral squamous cell carcinoma (OSCC) patients and reinforces the role of growth factors and proliferation-inducing cytokines. Patients with stage III or IV oral cavity squamous cell carcinoma (OSCC) were recruited and had blood drawn before the administration of any kind of treatment. Blood from healthy donors was used as a control. Plasma was separated and stored at -80°C until the concentrations of 48 factors were measured with a multiplex bead assay kit. The distributions between Healthy Donors (n = 7) and OSCC patients (n = 17) were compared using Mann-Whitney U tests. Differentially expressed factors (adjusted p-value < 0.05) were selected for multiple Spearman correlation

analysis. **(A)** Correlation matrices represent significant (p-value < 0.05) interactions between the selected factors. Correlations with p-values above 0.05 were shaded, while stars indicate distinct p-value thresholds. Red squares represent positive interactions, and blue squares represent negative interactions. **(B)** Exclusive interactions are shown for Healthy Donors (orange; n = 7) and OSCC patients (blue; n = 17). **(C)** Weighted relative interactions of each functional group (as described in **Supplementary Table 3**) are shown for Healthy Donors and OSCC patients in decreasing order of proportional interactions.

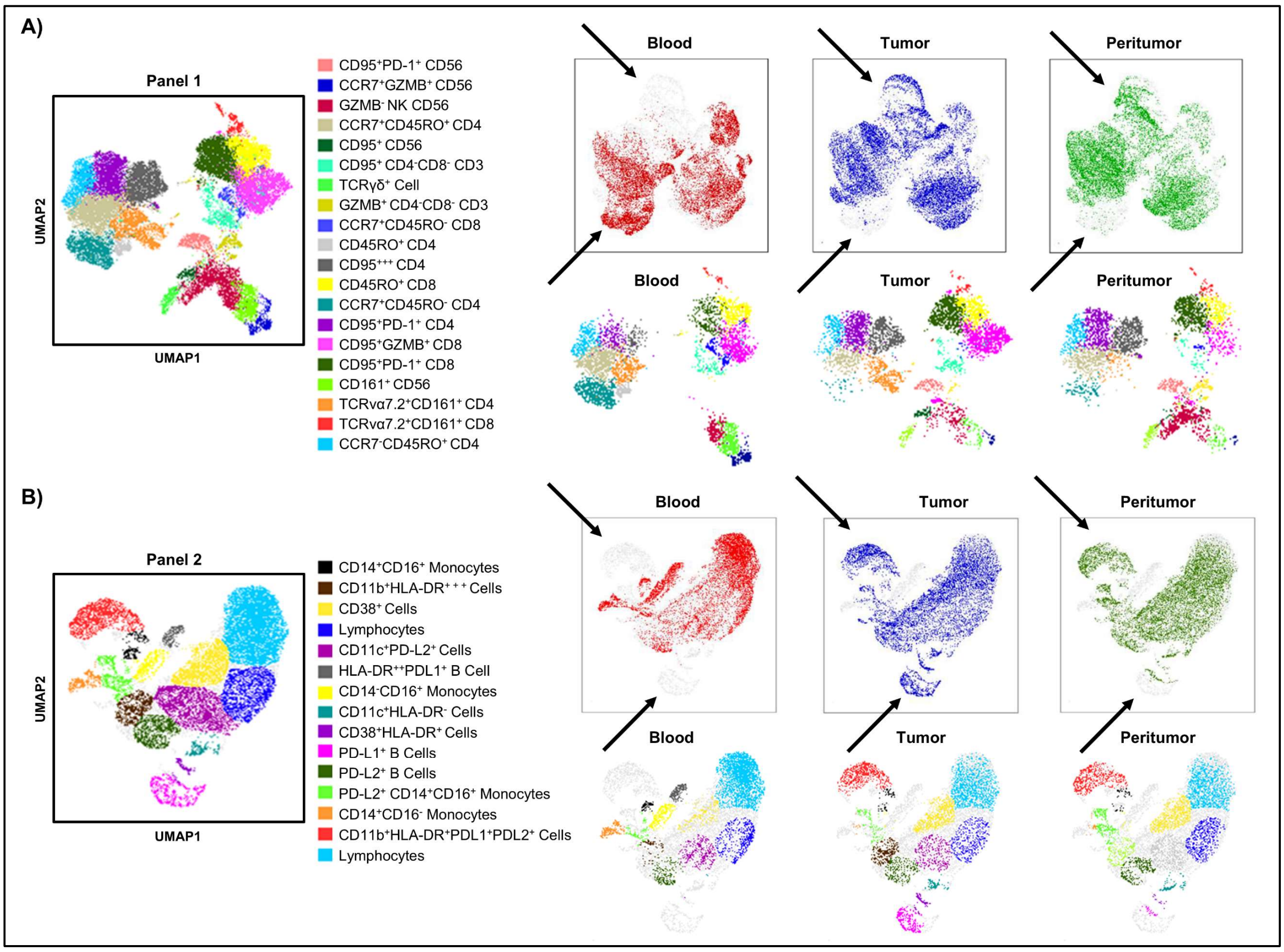

Supplementary Figure 4. Unsupervised cytometry maps compartment-specific clusters across lymphoid and myeloid lineages. Patients with stage III or IV oral squamous cell carcinoma (OSCC) were recruited and had blood drawn before the administration of any kind of treatment. Peripheral Blood Mononuclear Cells (PBMC) were obtained through density gradient centrifugation and assessed for immune marker expression by flow cytometry. Cells from tumor and peritumor were obtained through a dissociation protocol. Cytometry staining was done on the same day the samples were collected. At least 2000 events were concatenated to perform a Uniform Manifold Approximation and Projection (UMAP), followed by PhenoGraph algorithm to identify the major cell populations and their relative distributions based on compartment (blood, peritumor or tumor). **(A)** Representation of lymphocyte populations based on Panel 1 markers. **(B)** Representation of lymphocyte and monocyte populations based on Panel 2 markers. Arrows point to populations of interest that might present differential expression in different compartments. Panel information can be found in **Supplementary Table 1**.

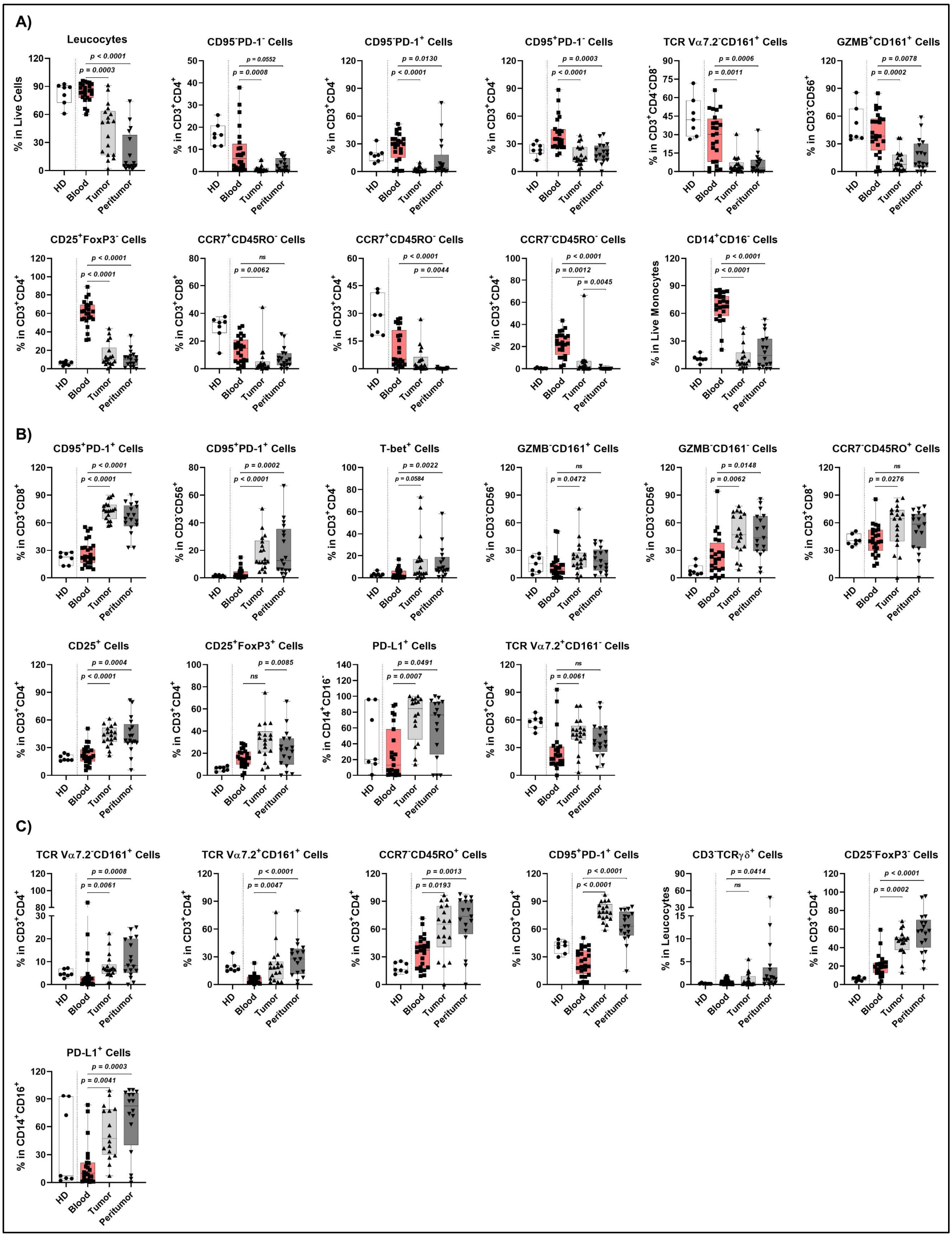

Supplementary Figure 5. Unpaired evaluation of immune cell subpopulations across blood, peritumor and tumor. Patients with stage III or IV oral cavity squamous cell carcinoma (OSCC) were recruited and had blood drawn before treatment. Peripheral Blood Mononuclear Cells (PBMCs) were obtained through density-gradient centrifugation and assessed for immune marker expression by flow cytometry. Cells from tumor and peritumor were obtained

through a dissociation protocol, and cytometry staining was performed on the day of sample collection. **(A)** Populations with higher frequency in blood samples. **(B)** Populations with higher frequency in peritumor samples. **(C)** Populations with higher frequency in tumor samples. Comparisons were performed using Kruskal-Wallis test with Dunn's post-hoc. The analysis used all available samples, with up to 24 blood,18 tumor and 17 peritumor samples. PBMCs from healthy donors (HD; n = 7) were used as a reference group, but statistical comparisons involved only data from patients. Graphs show median frequencies (% of parent population), interquartile ranges, and minimum and maximum values. Benjamini-Hochberg False Discovery Rate-adjusted p-values are shown. Population names are shown above each graph, and the y-axis indicates the parent population.

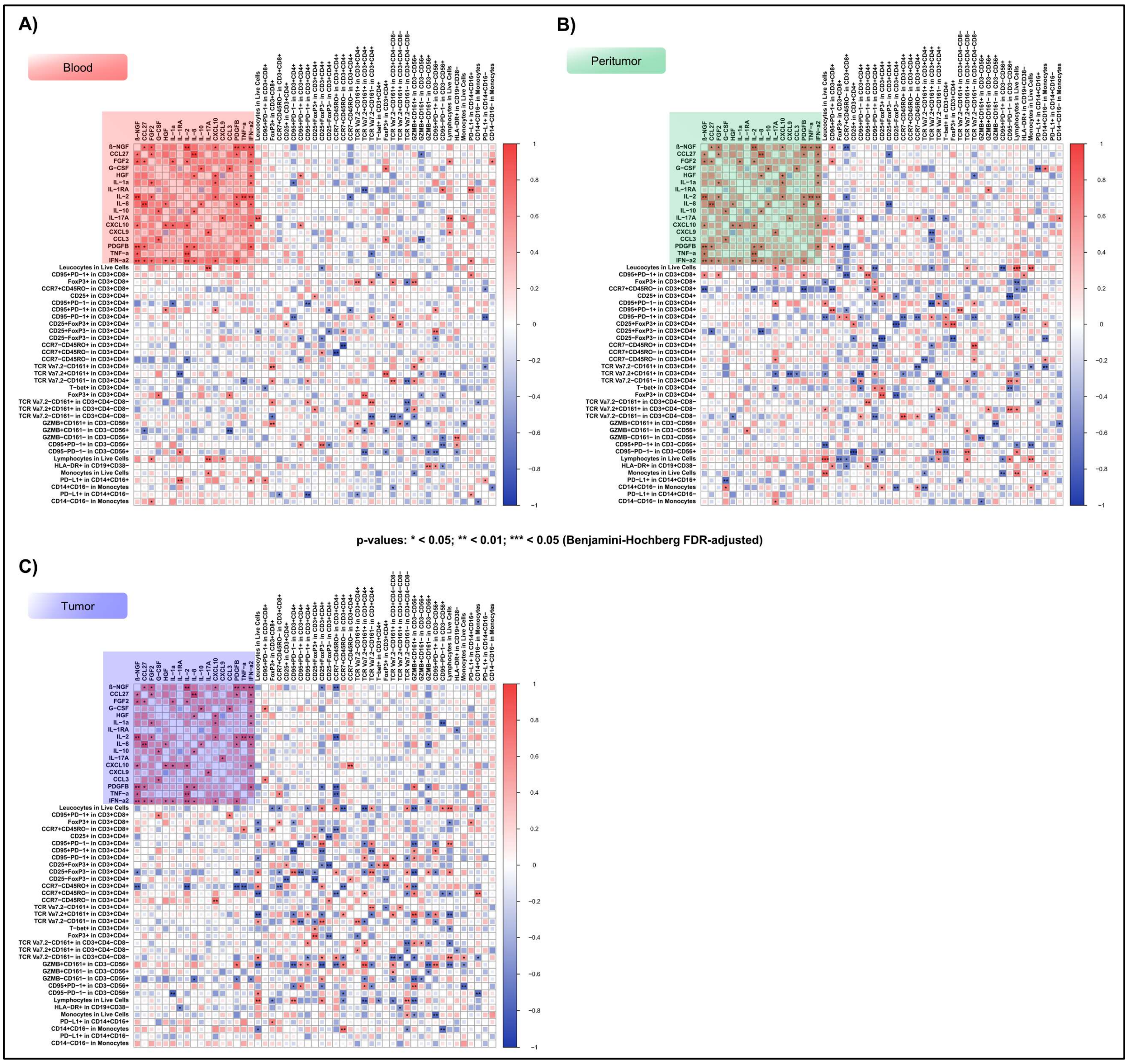

Supplementary Figure 6. Within-compartment immune interactions differ across blood, peritumor and tumor. Patients with stage III or IV oral squamous cell carcinoma (OSCC) were recruited and had blood drawn before the administration of any kind of treatment. Peripheral Blood Mononuclear Cells (PBMC) were obtained through density gradient centrifugation and assessed for immune marker expression by flow cytometry. Differentially expressed factors (considering all soluble immune mediators and differentially expressed cell populations from **Figures 1**, **3**) were selected for multiple Spearman's correlation analysis. **(A-C)** Correlation matrices depicting all interactions between different factors in blood **(A)**, peritumor **(B)** and tumor **(C)**. Correlations with a p-value above 0.05 were shaded, while stars were used to identify distinct thresholds of p-values. Red squares represent positive interactions, and blue squares represent negative interactions. A color-shaded box was added where the soluble factor's correlations grouped to facilitate the observation of other interactions.

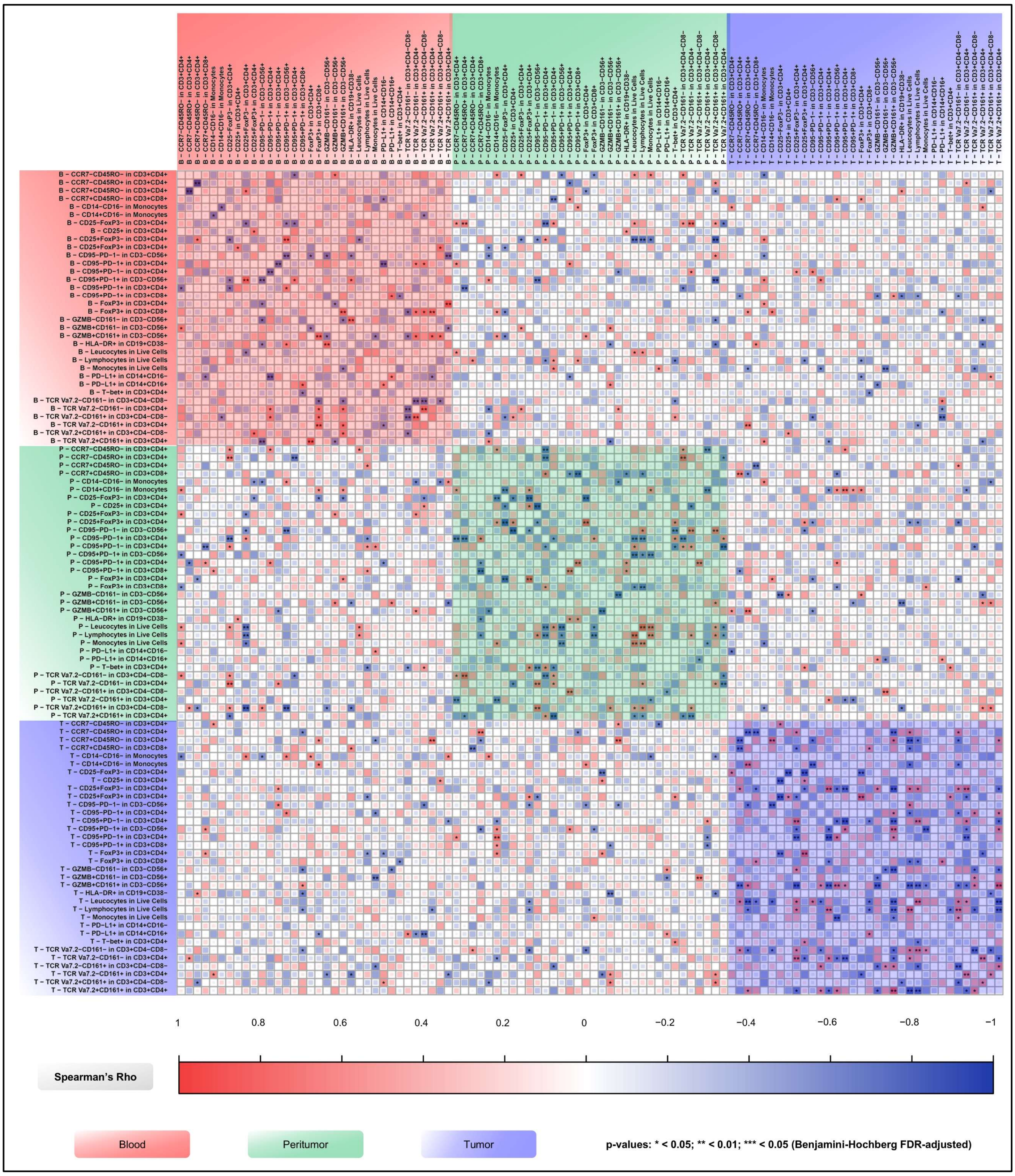

Supplementary Figure 7. Same-population cross-compartment correlations are consistent with phenotype shifts associated with tissue localization. Patients with stage III or IV oral squamous cell carcinoma (OSCC) were recruited and had blood drawn before the administration of any kind of treatment. Peripheral Blood Mononuclear Cells (PBMC) were obtained through density gradient centrifugation and assessed for immune marker expression by flow cytometry. Tumor and peritumor cells were obtained through a tissue dissociation protocol. Differentially expressed cell populations identified in **Figure 3** (p-value < 0.05) were selected for multiple Spearman's correlation analysis (n = 14). Correlation matrices depict all interactions between different factors; correlations with a p-value above 0.05 were shaded.

Red squares represent positive interactions, and blue squares represent negative interactions. A color-shaded box was added where the cell populations' correlations grouped to facilitate the observation of other interactions.
